# Current and potential roles of immuno-PET/-SPECT in CAR T-cell therapy

**DOI:** 10.3389/fmed.2023.1199146

**Published:** 2023-06-27

**Authors:** Aditi Mulgaonkar, Durga Udayakumar, Yaxing Yang, Shelby Harris, Orhan K. Öz, Praveen Ramakrishnan Geethakumari, Xiankai Sun

**Affiliations:** ^1^Department of Radiology, University of Texas Southwestern Medical Center, Dallas, TX, United States; ^2^Advanced Imaging Research Center, University of Texas Southwestern Medical Center, Dallas, TX, United States; ^3^Section of Hematologic Malignancies/Transplant and Cell Therapy, Division of Hematology-Oncology, University of Texas Southwestern Medical Center, Dallas, TX, United States

**Keywords:** immuno-PET, immuno-SPECT, cell therapy, CAR T-cell therapy, tumor microenvironment

## Abstract

Chimeric antigen receptor (CAR) T-cell therapies have evolved as breakthrough treatment options for the management of hematological malignancies and are also being developed as therapeutics for solid tumors. However, despite the impressive patient responses from CD19-directed CAR T-cell therapies, ~ 40%−60% of these patients' cancers eventually relapse, with variable prognosis. Such relapses may occur due to a combination of molecular resistance mechanisms, including antigen loss or mutations, T-cell exhaustion, and progression of the immunosuppressive tumor microenvironment. This class of therapeutics is also associated with certain unique toxicities, such as cytokine release syndrome, immune effector cell-associated neurotoxicity syndrome, and other “on-target, off-tumor” toxicities, as well as anaphylactic effects. Furthermore, manufacturing limitations and challenges associated with solid tumor infiltration have delayed extensive applications. The molecular imaging modalities of immunological positron emission tomography and single-photon emission computed tomography (immuno-PET/-SPECT) offer a target-specific and highly sensitive, quantitative, non-invasive platform for longitudinal detection of dynamic variations in target antigen expression in the body. Leveraging these imaging strategies as guidance tools for use with CAR T-cell therapies may enable the timely identification of resistance mechanisms and/or toxic events when they occur, permitting effective therapeutic interventions. In addition, the utilization of these approaches in tracking the CAR T-cell pharmacokinetics during product development and optimization may help to assess their efficacy and accordingly to predict treatment outcomes. In this review, we focus on current challenges and potential opportunities in the application of immuno-PET/-SPECT imaging strategies to address the challenges encountered with CAR T-cell therapies.

## Introduction

In recent years, strategies leveraging the human immuno-surveillance system to achieve complete remission of cancer have revolutionized the landscape of cancer immunotherapy. For instance, cytotoxic lymphocytes and natural killer cells, key players in the armor of the human immune system, have been employed to mediate immune surveillance. This approach takes advantage of their antitumor effector functions via distinct mechanisms, including (a) granule exocytosis resulting in the release of perforin and granule-associated enzymes (granzymes) and (b) release of exosomes containing Fas ligand (FasL) and tumor necrosis factor (TNF)-related apoptosis-inducing ligand (TRAIL), leading to the predominantly programmed apoptotic tumor cell death process (1). Currently, a solid foundation has been laid by the successes of T-cell-based cancer therapies in patients with metastatic cancers (2–4) as well as in patients with malignancies that have relapsed after, or were refractory to, the initial conventional treatments (5, 6). The redesign of a patient's own tumor-infiltrating lymphocytes (TILs) as cancer therapeutics, termed “adoptive cell therapy,” has garnered increasing interest in the field of immunotherapy since the 1960s (7). Efforts over the past three decades in adoptive T-cell therapy have resulted in the establishment of three types of cell therapies, namely, TIL therapies, engineered T-cell receptor (TCR) therapies, and chimeric antigen receptor (CAR) T-cell therapies (8, 9). In general, TIL cell therapies have achieved success in the effective management of melanomas (10), although the time required for donor cell expansion is a limiting factor. Moreover, factors such as T-cell exhaustion and T-cell dysfunction in the donor samples/starting material significantly impact the quality of the final products. More recently, the field has evolved with the sophisticated genetic engineering of peripheral T lymphocytes. This approach enables the design of superior antigen-targeted CAR T-cell therapies capable of effective tumor antigen binding for T-cell activation and proliferation independent of the major histocompatibility complex (MHC). Notably, the introduction of co-stimulatory domains in second-generation CAR T-cell therapy has enabled improved T-cell proliferation and persistence. Due to its high specificity toward a broader spectrum of membrane-expressed targets, CAR T-cell therapies have undergone significant translational development for treating cancers beyond B-cell malignancies. This has also prompted pharmaceutical companies to proceed toward commercialization of these therapies (11, 12).

In a Phase II single-cohort, multi-center global trial, the investigational Cluster of Differentiation 19 (CD19)-directed CAR T-cell therapeutic CTL019 (Tisagenlecleucel, Kymriah^®^, Novartis) demonstrated high response rates with an overall remission rate of 81% within 3 months and overall survival of 76% at 12 months in pediatric and young adult patients with relapsed/refractory (R/R) B-cell acute lymphoblastic leukemia (B-ALL) (13). This treatment received “*breakthrough therapy status*” and approval from the United States Food and Drug Administration (US-FDA) in 2017 for treating adult and pediatric R/R B-ALL (14). In yet another pivotal global Phase II trial with CTL019, a best overall response rate of 52% was observed in adult patients with R/R diffuse large B-cell lymphoma (LBCL), with an estimated 65% rate of relapse-free survival 12 months post initial response (15). Another successful trial was with axicabtagene ciloleucel (axi-cel, Yescarta^®^, Gilead), an autologous anti-CD19 CAR T-cell therapy. This received US-FDA approval in 2017 (16) for the treatment of adults with R/R LBCL after two or more lines of systemic therapies [ZUMA-1 trial (17)]. Recently, it has been extended for use in R/R follicular lymphoma (FL) and in earlier lines of therapy in LBCL with manageable adverse events (ZUMA-5, ZUMA-7, and ZUMA-12 trials) (17–19). Other approved CD19-directed CAR T-cell therapies include lisocabtagene maraleucel [liso-cel, Breyanzi^®^, Bristol Myers Squibb (BMS)] (20, 21) for R/R LBCL in the second/third-line setting and brexucabtagene autoleucel (brexu-cel, Tecartus^®^, Gilead) for R/R mantle cell lymphoma (MCL) (22) and adult B-ALL (23). Two CAR T-cell therapies targeting the B-cell maturation antigen (BCMA), idecabtagene vicleucel (idecel, Abecma^®^, BMS) (24) and ciltacabtagene autoleucel (ciltacel, Carvykti^®^, Janssen) (25), have been approved in R/R multiple myeloma. Additionally, other investigational CAR T-cell therapies targeting a variety of antigens are showing promising results in Phase I/II trials across the spectrum of hematologic malignancies (26–30). In solid malignancies, however, CAR T-cell therapies have encountered several hurdles resulting from tumor target heterogeneity, tumor penetration issues, and the immunosuppressive tumor microenvironment (TME). However, there are several ongoing early-phase clinical trials working on advancing these therapies to solid tumors such as brain, pulmonary, gastrointestinal, renal, hepatic, thoracic, ovarian, and prostate cancers (31, 32).

While overall response rates to CAR T-cell therapies have been impressive, challenges remain including manufacturing difficulties as a result of dysfunctional T-cells and expansion times. Toxicities regarded as a “class-effect” with these therapies are also of major concern. These include life-threatening forms of toxicity such as cytokine release syndrome (CRS), immune effector cell-associated neurotoxicity syndrome (ICANS), and others associated with “on-target, off-tumor” recognition and anaphylaxis (33–35). While the exact mechanisms underlying these adverse events (AEs) remain to be elucidated, the likely cause is thought to be a cytokine surge, which occurs with the immunological-activation cascade triggered by effector mechanisms of CAR T-cells. Another significant challenge is the recurrence of cancer in a significant proportion of patients after CAR T-cell therapy. Cancer relapses may occur due to varied combinations of intrinsic and/or extrinsic factors in the TME during and post CAR T-cell therapy. These include target antigen loss/mutations, peripheral and tumor-infiltrating T-cell exhaustion or senescence, immunogenicity-reducing alterations in the tumor mutational burden, and tumor progression due to an immunosuppressive TME (5, 34–37). Thus, it is imperative to identify such events when they occur to enable timely changes in treatment. Currently, efforts have been made to identify physiological biomarkers with prognostic implications, which may enable better management of these cell therapies (38).

Conventional pathology assays used in clinical practice for cancer management [e.g., immunohistochemistry, flow cytometry, enzyme- or polymerase chain reaction (PCR)-based assays] are limited by the availability of biopsy tissues. Additionally, the results obtained from these biopsied tumor samples suffer from spatial limitations. Given that the TME is physiologically and genetically heterogeneous, a tumor sample biopsied at any given location may not be representative of the characteristics of the entire primary tumor or distant metastases (39). Moreover, such tissue-based invasive techniques lack the “real-time” detection capabilities to capture dynamic variations in target expression during therapy or when AEs occur.

Together with their inherent capabilities for deep tissue penetration, “real-time” whole-body imaging, and high sensitivity for quantification, the non-invasive radionuclide imaging modalities of positron emission tomography (PET) and single-photon emission computed tomography (SPECT) are now enabling the development of technologies to address these challenges when equipped with radiolabeled monoclonal antibodies (mAbs) or engineered mAb fragments (40–45). Especially in the case of solid tumors, where CAR T-cell therapies suffer from challenges associated with infiltration into an immunosuppressive TME, these molecular imaging modalities may find application for the detection of CAR T-cell distribution, expansion, and clearance throughout the therapeutic regimen (46).

In this review, we discuss opportunities for immuno-PET/-SPECT imaging strategies to address the challenges encountered with these CAR T-cell therapies, and thereby to act as an important guidance tool for optimal therapeutic management.

## Current advances in immuno-PET/-SPECT imaging methods and their potential to address the challenges with CAR T-cell therapy

The most commonly used PET radiotracer is 2-deoxy-2-[^18^F]fluoroglucose ([^18^F]FDG). A glucose analog, [^18^F]FDG is taken up by tumor cells via membrane-bound glucose transporters, where it is phosphorylated into [^18^F]FDG-6-phosphate and trapped in cells. This trapped metabolite uptake can be quantified by PET using a standardized uptake value (SUV) and correlates with disease severity. PET with [^18^F]FDG is used in clinical practice across a wide range of cancers for initial tumor diagnosis and staging and for the longitudinal assessment of therapy response (47–49). However, since [^18^F]FDG is primarily a metabolic radiotracer that measures elevated glycolysis, it cannot differentiate malignancies from co-existing non-malignant inflammatory conditions caused by rheumatological diseases, infections, or AEs encountered with cell-based immunotherapies (50). Owing to the increasing applications of inherently immunogenic CAR T-cell therapies for cancers, the development of immuno-PET/-SPECT strategies is warranted to delineate the interactions between malignancies and these supraphysiological immunological processes.

**Figure 1:**
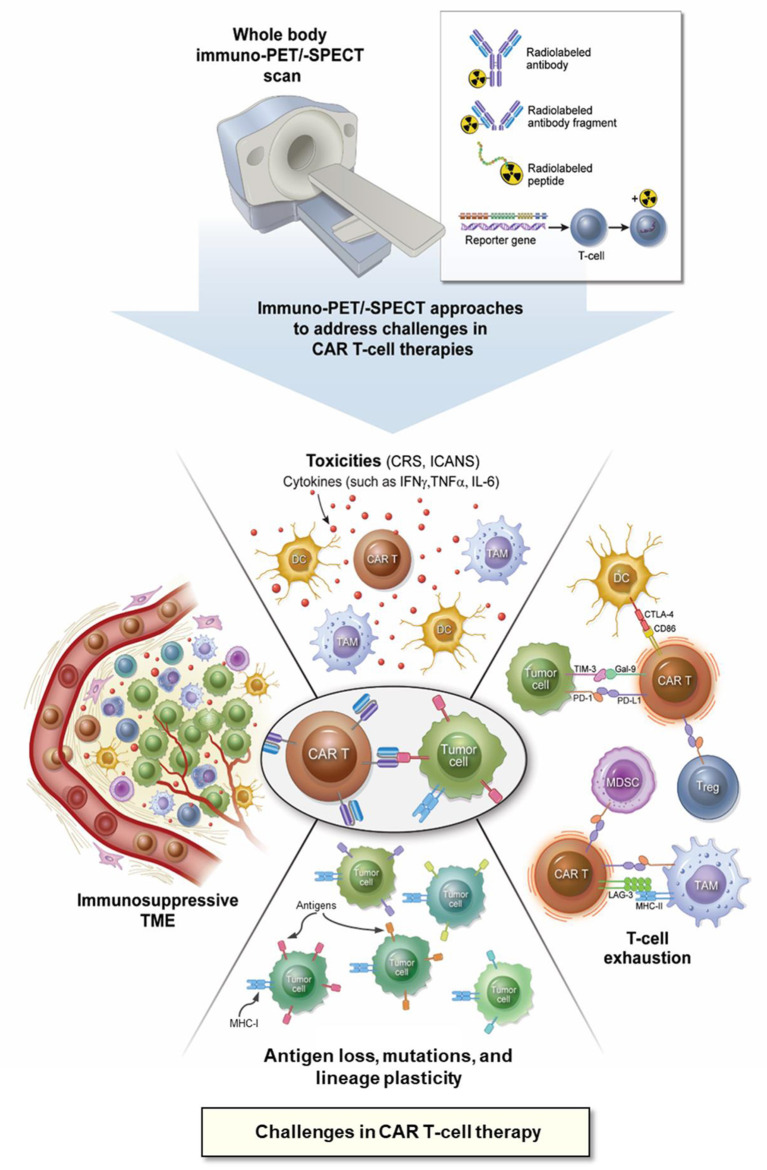
Application of immuno-PET/-SPECT imaging approaches to address the challenges with CAR T-cell therapies. CAR, chimeric antigen receptor; CD, cluster of differentiation; CRS, cytokine release syndrome; CTLA-4, cytotoxic T lymphocyte antigen-4; DC, dendritic cell; Gal-9, galectin 9; ICANS, immune effector cell-associated neurotoxicity syndrome; IFNγ, interferon gamma; IL-6, interleukin 6; immuno-PET/-SPECT, immunological positron emission tomography or single-photon emission computed tomography; LAG-3, lymphocyte activation gene 3 protein; MDSC, myeloid-derived suppressor cell; MHC, major histocompatibility complex; PD-1, programmed cell death protein 1; PD-L1, programmed cell death protein ligand 1; TAM, tumor-associated macrophage; TIM-3, T-cell immunoglobulin and mucin domain protein 3; TME, tumor microenvironment; TNFα, tumor necrosis factor alpha, T_reg_, regulatory T-cell.

Early identification of the dominant resistance mechanisms within the heterogeneous and often immunosuppressive TME or the occurrence of toxic events associated with CAR T-cell therapies is essential to ensure successful therapeutic interventions. Additionally, as novel CAR T-cell therapies are developed, an assessment of their *in vivo* pharmacokinetics (biodistribution and “homing” to tumors, expansion, and clearance or potential destruction) is critical for reliable determination of their efficacy and prediction of the therapeutic outcome (46, 51). Combining the intrinsic sensitivity of PET/SPECT with the superior targeting specificity offered by mAbs (41, 42, 52, 53), immunological PET/-SPECT (immuno-PET/-SPECT) can be leveraged or tailored to address the following challenges encountered with CAR T-cell therapies (Figure 1).

### Antigen loss

Antigen escape is one of the most commonly encountered mechanisms of cancer resistance to CAR T-cell therapy. It usually occurs in cases of cancer relapse after complete remission in patients, resulting in a phenotypically similar disease, but with either complete loss or downregulation of the target antigen expression. Consequently, the relapsed disease becomes non-responsive to the CAR T-cell treatment. For example, while remarkable response rates (70%−90%) have been observed for B-ALL in patients treated with CD19-directed CAR T-cell therapies such as CTL019 (54) in early-stage trials, follow-up studies have reported leukemia recurrence in ~50% of patients, typically 1 year post-therapy (54–56). Such cancer relapses associated with the loss of CD19 antigen have been reported in both children (~18%−25%) and in adult populations (~7%−9%) in Phase I studies (6, 54, 57–59). Given the multitude of clinical studies being conducted using CD19-directed CARs and bispecific T-cell engagers (BiTEs) (60), decreased/loss of antigen expression by R/R tumors may be attributed to various mechanisms, depending on the subject pool of a given study. Persistent immune pressure from CAR T-cell therapies may result in selective progression of tumor cells with genetic alterations in the CD19 protein, enabling antigen escape from CD19-directed CAR T-cell therapy (61–63). For example, in one reported study, whole-exome DNA- and RNA-sequencing analysis of baseline vs. post-relapse CD19-negative patient samples of R/R B-ALL demonstrated acquired frameshift mutations in CD19 exons 2–5. This likely resulted in a truncated protein sequence lacking transmembrane anchorage, leading to antigen escape (62). Furthermore, this study found that the allelic frequencies of the mutations correlated with the CD19-negative cells by flow cytometry and concluded that homozygous bi-allelic mutations (loss of heterozygosity) in CD19 are the primary resistance mechanism for CD19-negative relapse. Similar mechanisms for such inherited molecular resistance as a result of target antigen modulation have been observed for other targets of hematological malignancies, including CD22 in LBCLs (26), BCMA in myelomas (64), and even in solid tumors such as glioblastomas [epidermal growth factor receptor (EGFR) (65) and interleukin 13 receptor alpha 2 (IL13Rα2) (66)]. “Lineage switch” is another poorly understood mechanism of resistance to CAR T-cell therapy. In lineage switch, hematological cancer cells can undergo intrinsic changes to relapse as a clonally similar but phenotypically different cancer sub-type (67, 68). Such lineage plasticity is often encountered in pediatric and infant patients with refractory B-ALLs expressing mixed-lineage leukemia rearrangements (MLL-r). In such cases, the leukemia cells “switch” lymphoid physiological markers to become cells of a myeloid phenotype (68, 69). Relapses associated with MLL-r to acute myeloid leukemia (AML) have been seen with CD19-directed CAR T-cell therapy as well as BiTEs; however, other cases of phenotypic variation have also been observed (70–74). Other reported mechanisms for antigen reduction and escape include a phenomenon known as “trogocytosis,” whereby the CAR T-cells can strip the neighboring lymphoma cells of their target protein and incorporate it into the plasma membrane of the CAR T-cells, resulting in reduced surface target density. Trogocytosis may also result in “fratricide” by causing CD19^+^ T-cell death and promoting T-cell exhaustion (75–77). Such conditions could negatively impact the efficacy of CAR T-cell therapies (75, 76, 78, 79).

**Figure 2:**
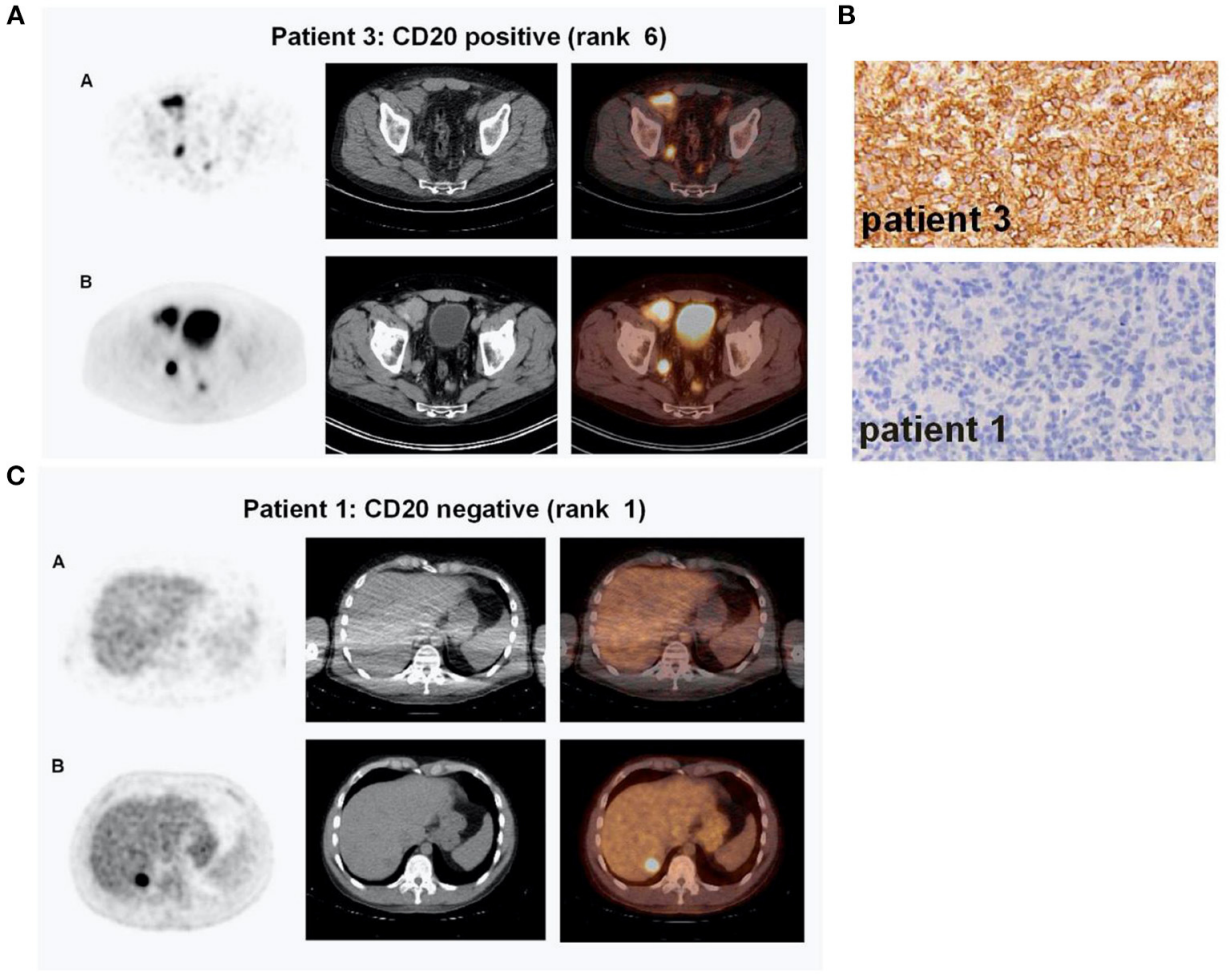
Immuno-PET imaging with [^89^Zr]Zr-Rituximab in patients with DLBCL treated with a therapeutic dose of rituximab. **(A)** Immuno-PET imaging shows intense tumor uptake (top panel) concordant with CD20-positive IHC staining of the inguinal lymph node biopsy sample (**B**, top panel). Corresponding [^18^F]FDG-PET images are shown in the bottom panel **(A). (C)** CD20-negative tumor shows no appreciable uptake of [^89^Zr]Zr-Rituximab (top panel) concordant with IHC staining of the biopsy (**B**, bottom panel). In contrast, the tumor exhibits a focal spot on [^18^F]FDG-PET images (**C**, bottom panel). Shown in **(A)** and **(C)** are attenuation-corrected PET, low-dose CT, and fused PET/CT images from left to right. Reproduced with slight format modifications from the open-access article in ref. (81) under the Creative Commons license (http://creativecommons.org/licenses/by/4.0/).

For the assessment of transient modulation, loss in antigen expression, or loss of function due to antigen mutation, immuno-PET/-SPECT imaging approaches using radiotracers derived from specific mAbs against cancer-overexpressing targets would be extremely valuable (Supplementary Table S1). Indeed, several surface target antigens in hematological malignancies have been considered for such imaging evaluations, including CD19, CD22, CD20, BCMA, and CD38 (53, 80). For example, immuno-PET imaging with a zirconium-89 ([^89^Zr])-labeled anti-CD20 mAb, [^89^Zr]Zr-DFO-rituximab, was reported in five patients with DLBCL (81). A correlation was found in this study between the imaging signal and CD20 expression measured by immunohistochemical (IHC) staining (Figure 2). More recently, a case report on immuno-PET with the same mAb but labeled with copper-64 (^64^Cu), [^64^Cu]Cu-DOTA-rituximab, demonstrated higher sensitivity than [^18^F]FDG-PET for imaging lymphoma tumors in two patients (82). Interestingly, although CD19 is an ideal target for CD19-directed CAR T-cell therapy in B-cell malignancies, to the best of our knowledge, there are no reports yet on CD19-targeted immuno-PET/-SPECT imaging to address antigen loss. However, a CD19-targeted immuno-PET method with [^64^Cu]Cu-CD19-mAb, a murine anti-CD19, has been reported to produce a PET signal correlating with B-cell distribution in the central nervous system (CNS) in an experimental autoimmune encephalomyelitis mouse model (83). Recently, with increasing efforts directed toward the application of CAR T-cell therapies for treating solid tumors, the target spectrum for CAR T-cell engineering has broadened considerably. Apparently, reported immuno-PET strategies using specific mAbs targeting solid tumor surface antigens, including EGFR [[^89^Zr]Zr-cetuximab (84), [^89^Zr]Zr-panitumumab (85, 86)], human epidermal growth factor receptor 2 (HER2) [[^89^Zr]Zr-trastuzumab (87–89), [^111^In]In-pertuzumab (90)], prostate-specific membrane antigen (PSMA) [[^89^Zr]Zr-J591 (91)], vascular endothelial growth factor (VEGF) [[^89^Zr]Zr-bevacizumab (92)], can be leveraged to non-invasively monitor antigen expression throughout the duration of CAR T-cell therapy (93). Furthermore, immuno-PET imaging methods may play an instrumental role in the detection of transient shifts in antigen expression, especially in ambiguous-lineage hematological cancers. For instance, longitudinal tracking of lymphoid lineage-specific surface antigens, such as CD19, CD20, CD3, and CD4, by specific mAbs or their fragments may enable the detection of resistance induced by “lineage switching” in rare high-risk ambiguous-lineage leukemias [such as in the case of mixed-phenotype acute leukemia (MPAL) switching to B- or T-cell ALL or vice versa (74, 94), or in MLL switching to AML (67)]. Of course, such rare malignancies often involve variations in multiple lymphoid or myeloid lineage surface markers, necessitating additional investigations on a case-by-case basis regarding the practicality of using immuno-PET/-SPECT imaging as a guidance tool for their management. It is noteworthy that immuno-SPECT is capable of simultaneous imaging of multiple surface markers if the corresponding surface antigens are targeted with mAbs labeled with radionuclides emitting differentiable gamma energies. Indeed, SPECT imaging with dual radiotracers has been reported in clinical applications (95–99) and preclinical studies (100–102). To date, non-invasive assessment of multiple biomarkers/molecular processes via a single immuno-SPECT scan has been made possible by the use of solid-state cadmium zinc telluride (CZT) gamma detectors, which offer higher energy resolution and detection sensitivity than the conventional sodium iodide (NaI) detectors, as well as the current implementation of novel image processing algorithms (96, 103).

**Figure 3:**
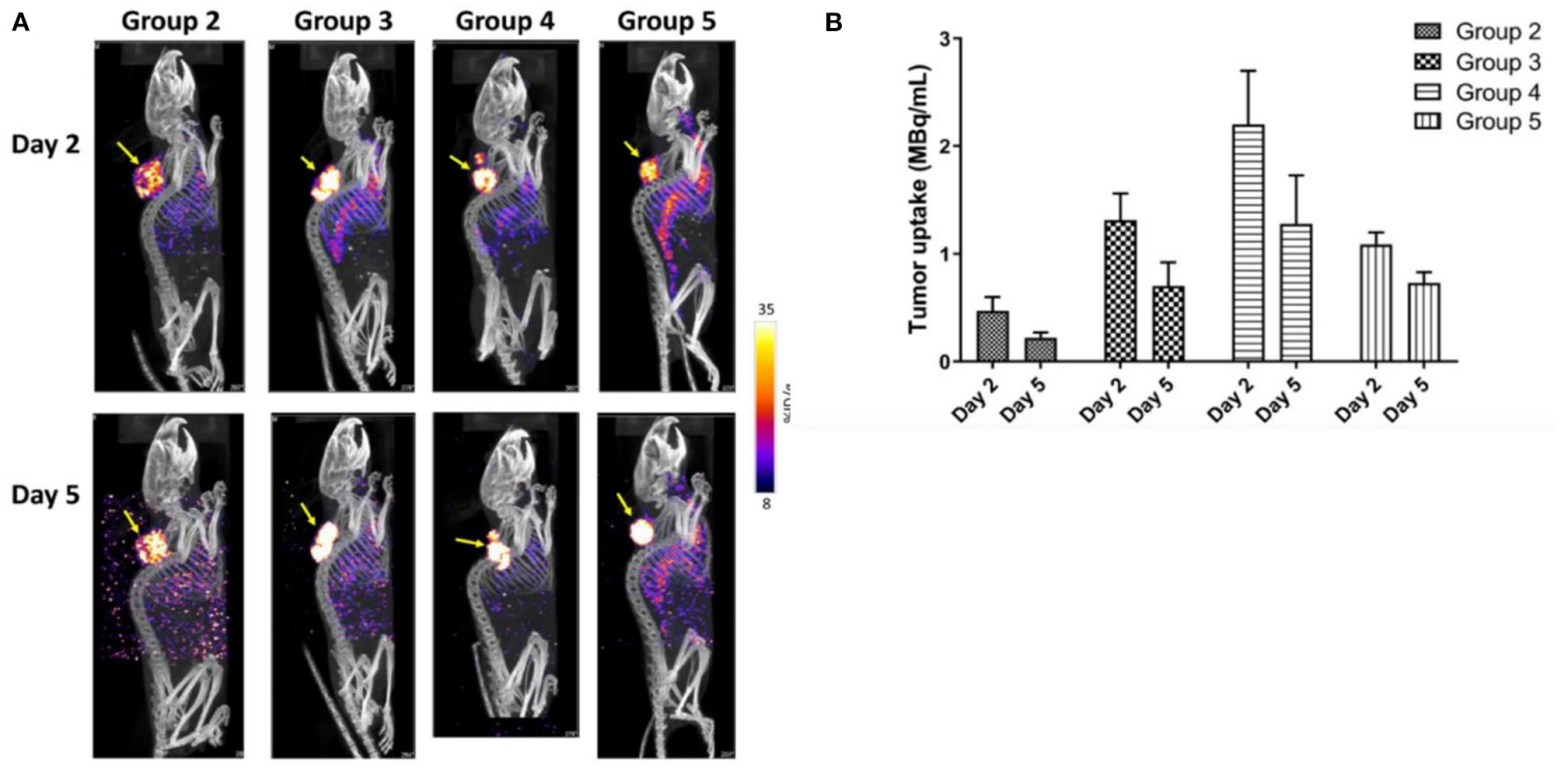
Immuno-SPECT/CT imaging of HCC1954 HER2^+^ tumor-bearing mice injected with [^67^Cu]Cu-NOTA-Pertuzumab. **(A)** Representative maximum intensity projection (MIP) of SPECT/CT images in mouse groups as indicated at days 2 and 5 post-treatment (yellow arrows indicate the tumors). **(B)** Actual radioactivity concentration in tumors (MBq/ml) on days 2 and 5 (without decay correction). Reproduced from the open access article in ref. (42) under the Creative Commons license (http://creativecommons.org/licenses/by/4.0/).

Given the long circulation half-lives of antibodies in the blood, immuno-PET/-SPECT imaging requires a half-life-matched radionuclide to label a mAb or an engineered fragment. Recently, ^89^Zr with a half-life (t_1/2_) of 3.27 days and ^64^Cu (t_1/2_ = 12.7 h) have gained popularity for labeling of antibodies and fragments because they can be produced in-house by a biomedical cyclotron equipped with solid-target capability. In terms of imaging sensitivity, immuno-PET is preferred over immuno-SPECT (53), while the latter can be readily performed when a radioimmunotheranostic agent is used. The decay of therapeutic radionuclides, such as lutetium-177 (^177^Lu, t_1/2_ = 6.65 days) and copper-67 (^67^Cu, t_1/2_ = 2.57 days), usually involves gamma rays that can be imaged with a SPECT scanner (42, 46). As shown in Figure 3, we reported on a study with [^67^Cu]Cu-pertuzumab in murine HER-2-positive xenografts demonstrating the specificity of immuno-SPECT imaging as well as the improved therapeutic efficiency resulting from the increase in molar activity of the radiotracer (42). Of note, if imaging sensitivity is desired, the therapeutic radionuclides can be replaced or paired with proper positron-emitting radionuclides [e.g., iodine-131 (^131^I, t_1/2_ = 8 days) with iodine-124 (^124^I, t_1/2_ = 4.2 days), ^67^Cu with ^64^Cu, yttrium-90 (^90^Y, t_1/2_ = 2.7 days) with yttrium-86 (^86^Y, t_1/2_ = 14.7 hours), and ^177^Lu (^177^Lu, t_1/2_ = 6.6 days) with gallium-68 (^68^Ga, t_1/2_ = 68 min)] for immuno-PET without drastically altering the targeting properties and *in vivo* kinetics of the radioimmunotheranostic agents. Such theranostic agents may find a greater role in the non-invasive monitoring of dynamic changes in antigen levels for precision CAR T-cell therapies.

### T-cell exhaustion and senescence

T-cell exhaustion and senescence are two dysfunctional states that heavily influence cancer management and patient outcomes with CAR T-cell therapies (104–106). While T-cell senescence is often associated with aging, it also occurs in chronic infections and in certain cancers (107). T-cell senescence is characterized by events including telomere shortening (during cell division), phenotypic changes (loss of CD28 domain), and cell cycle arrest, where the T-cells are live and metabolically active, but incapable of further proliferation or differentiation. This results in the loss of naïve and effector T-cells and dysregulation of the immune system (108, 109). T-cell exhaustion is a “hypo-responsive” state attained by T-cells after losing their effector functions as a result of persistent activation by antigens in response to chronic infections or tumor progression. In acute infections, the effector T-cell (T_eff_) population is gradually deactivated after the antigen is cleared or destructed, with the retention of a functional memory phenotype (T_mem_). However, under conditions of persistent activation by CAR T-cell therapy, or during pathological chronic antigen stimulation, the CD8^+^ T_eff_ cell population eventually differentiates into an exhausted phenotype (T_ex_) characterized by a lack of further differentiation and loss of effector function (107, 110, 111). Notably, the immunosuppressive TME plays an important role in driving the T_eff_ population toward exhaustion and senescence (37, 105, 112). While senescent T-cells share overlapping phenotypes with exhausted T-cells, each has distinct mechanisms. Current studies indicate that while mitogen-activated protein kinase regulates T-cell senescence, T-cell exhaustion is mediated by inhibitory checkpoint proteins in the immunosuppressive TME and characterized by decreased cytokine secretion (106).

Some classic immune checkpoint receptors, which are T-cell exhaustion markers, include programmed cell death protein 1 (PD-1), lymphocyte activation gene 3 protein (LAG-3), T-cell immunoglobulin and mucin domain protein 3 (TIM-3), cytotoxic T lymphocyte antigen-4 (CTLA-4), B and T lymphocyte attenuator (BTLA), V-domain immunoglobulin-containing suppressor of T-cell activation (VISTA), and T-cell immunoglobulin and immunoreceptor tyrosine-based inhibitory motif domain (TIGIT) (69, 106, 107, 111, 113–115). Among the comprehensively studied proteins, binding of PD-1 to its ligand PD-L1 is reported to regulate T-cell immunosuppression by initiating the inhibitory downstream signaling of zeta-chain-associated protein kinase 70-extracellular signal-regulated kinase (ZAP70-ERK) and phosphatidylinositol-3 kinase-protein kinase B (PI3K-AKT) via the recruitment of Src homology 2 domain-containing tyrosine phosphatases 1 and 2 (SHP1 and SHP2), and can arrest T-cell proliferation via inhibition of cyclin-dependent kinases (106, 116). CAR T-cell therapies with CD28 stimulation domain (rather than 4-1BB) have shown more susceptibility to inhibition via the PD-1/PD-L1 checkpoint axis by direct inactivation of the CD28 signaling domain (106, 117–119). Interestingly, clinical trials combining immune checkpoint inhibitor (ICI) antibodies with CD19-directed CAR T-cell therapies (120–124) and novel CAR T-cell design strategies blocking the PD-1/PD-L1 interactions (119, 125–127) have demonstrated prolonged T-cell persistence and promising treatment outcomes.

**Figure 4:**
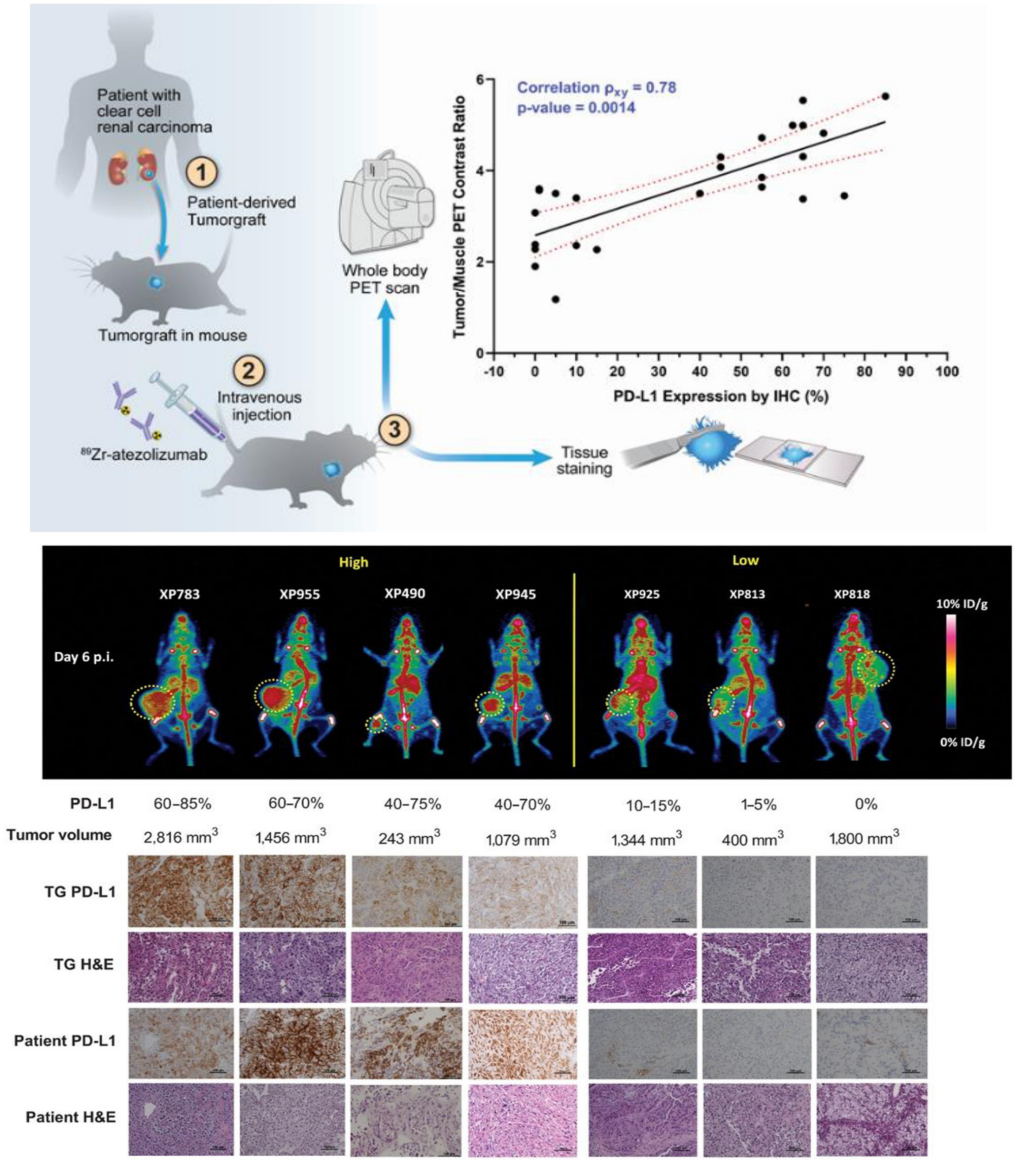
Integrated immuno-PET, IHC, and hematoxylin and eosin (H&E) images of mouse tumorgraft lines from corresponding patient tumors with high and low PD- L1 expression groups. **(Top)** Schematic representative of the workflow. **(Middle)** Representative whole-body MIP immuno-PET images (posterior view) of mice, one from each group. The corresponding PD-L1 expression ranges measured by IHC and the volume of the tumor in the mouse as indicated are shown below (*n* = 3–4 for each line; a single remaining XP258 mouse is not included). Tumors are indicated with a yellow lasso. **(Bottom)** PD-L1 IHC and H&E staining of the corresponding tumor tissues explanted from the TG models. Patient tumor samples shown as a reference. A part of this figure has been reproduced from the referenced article (41) under reuse permission from standard copyright in AACR journals for authors.

Thus far, immuno-PET has advanced to a point enabling imaging evaluation of T-cell exhaustion pathways and immunosuppressive biomarkers in the TME (Supplementary Table S2). Promising clinical data using radiolabeled intact mAbs targeting the inhibitory checkpoint proteins, such as PD-1 (128, 129), PD-L1 (41, 130–132), and CTLA-4 (133, 134), have fundamentally validated the immuno-PET approach. For instance, clinical studies with [^89^Zr]-labeled atezolizumab have demonstrated that ICI treatment outcomes can be better predicted with immuno-PET performed before ICI than with other tissue-based methods (e.g., ribonucleic acid (RNA)-sequencing or IHC) in three solid tumor types (triple-negative breast cancer, bladder cancer, and non-small cell lung cancer) (130). As shown in Figure 4, immuno-PET with this radiotracer has also shown potential for stratification of patients with renal cell carcinomas based on imaging-assessed PD-L1 expression in patient-derived tumor grafts and in a clinical report (41, 131). In addition to intact mAbs of PD-L1, immuno-PET with radiotracers derived from small adnectin proteins have also produced encouraging results in multiple clinical studies (135–137). More recently, alternative inhibitory immune checkpoint receptor/ligand pathways (e.g., TIGIT, LAG-3, and TIM-3) have been employed to design immunotherapies that can avert the toxicities associated with anti–PD-1/PD-L1 and anti-CTLA-4 ICIs and improve treatment efficacy as combination therapies. These immunotherapies can be readily adapted for immuno-PET imaging. Indeed, such imaging methods have demonstrated the capability to capture variation in these checkpoint proteins (138–141). For instance, with variable but high expression on TILs in solid tumors, TIGIT is also present on activated CD8^+^ T-cells, activated CD4^+^ regulatory T-cells, and natural killer (NK) cells (138, 142). This protein mediates inhibition of innate and adaptive immunity through inhibition of T-cell and NK cell immune responses (143, 144). Immuno-PET with TIGIT-specific [^64^Cu]Cu-TIGIT-mAb and [^89^Zr]Zr-TIGIT-mAb has demonstrated high specificity for TIGIT expression in xenograft (HeLa-TIGIT in nu/nu mice) and allograft (B16 melanoma in B6 mice) models (138). These results suggest the feasibility of utilizing immuno-PET for non-invasive patient stratification based on TIGIT expression for anti-TIGIT therapy. Such imaging methods hold great potential to guide combination ICI treatment (145) in order to overcome T-cell exhaustion during CAR T-cell therapies.

In contrast to the well-documented roles of T-cell exhaustion in cancer relapse/resistance against CAR T-cell therapies, the mechanisms of T-cell senescence mediated by the TME remain largely unknown (146, 147). The senescent T-cell phenotype is associated with substantial downregulation of CD28 and CD27 stimulatory markers and an increase in beta-galactosidase (SA-β-gal) activity (147, 148). Other T-cell senescence-associated markers include TIM-3, CD57, and killer cell lectin-like receptor subfamily G member 1 (KLRG-1) (149–152). Moreover, a unique senescence-associated secretory phenotype (SASP) has been reported with senescent T-cells; this phenotype generates large amounts of pro-inflammatory cytokines, such as interleukin 2 (IL-2), IL-6, IL-8, TNF-α, and interferon gamma (IFN-γ), in addition to the suppressive cytokines IL-10 and transforming growth factor β (TGF-β) (146, 153, 154). As such, PET has been employed to detect T-cell senescence via imaging of surrogate markers, such as TIM-3 (141), overexpression of SA-β-gal enzyme (155–157), IL-2 (158), TNF-α (159), and IFN-γ (160) (Supplementary Tables S2–S4).

There are several important factors to consider in the development of radiolabeled antibodies for immuno-PET/-SPECT imaging: for instance, the Fc-receptor interactions. Although these interactions may be advantageous for some therapeutic mAbs due to the resulting prolongation of their systemic half-lives and accentuation of their effector functions (161), they may expose patients to higher doses of radiation when radiolabeled for radiotherapy or imaging. In addition, such Fc interactions may compromise the desired antigen-targeted immuno-PET signal due to a high non-target uptake (162). To overcome this issue, it is plausible to silence the Fc domain (163) [e.g., through selection of mAbs such as atezolizumab (164)] or to use engineered mAb fragments (e.g., minibodies, diabodies, and BiTEs) (165). Other issues with this approach include the solubility of target antigens in plasma (166–168).

### CAR T-cell distribution

For manufacturing of CAR T-cell products, the recipient patient's own (autologous) T-cells are the preferred source in order to avoid the possibility of graft-vs.-host reactions due to the use of donor T-cells. However, adherence to this personalized adoptive cell therapy can jeopardize the extension of treatment benefits to a larger cohort of patients (126). In general, CAR T-cell therapies inherently suffer from manufacturing limitations associated with long production times due to the required T-cell selection and expansion processes to ensure a high-quality product (34). Moreover, most patients receiving these CAR T-cell therapies suffer from advanced cancers and have previously undergone conventional chemotherapies or other immunotherapies. Consequently, these patients may be lymphopenic with a high possibility of dysfunctional or exhausted T-cells (169). This can be a severe limiting factor for product development, significantly impacting the treatment outcome (170–172). To address these challenges, the development of allogeneic CAR T-cells from donors has gained impetus as an alternative strategy enabling a large scale universal production for “off-the-shelf” doses of the CAR T-cell product. Novel research strategies may also involve designing combination CAR T-cells and CAR T-cells with BiTE formulations (60, 126, 173). Moreover, “universal CARs” can be designed with dual targeting capabilities to overcome resistance to CAR T-cell therapy owing to the loss of a single antigen (174, 175). Nevertheless, the risks associated with potential toxicities, such as graft-vs.-host and autoimmunity reactions, require careful consideration in the context of such strategies (176). To avoid toxicities, standard-of-care CAR T-cell therapy protocols recommend the injection of only a limited number of CAR T-cells (~10^5^ to 10^6^ cells per kg of body weight) (177). With the capability for direct tracking of the *in vivo* dynamic distribution of CAR T-cells and TILs, immuno-PET/-SPECT may provide pivotal information for the optimization therapeutic outcomes and enable evaluation of therapy-induced alterations and detection of resistance mechanisms (43, 178). Because others have reviewed these approaches in detail (46, 51, 179), we provide only a few highlights below and in Supplementary Table S3.

**Figure 5:**
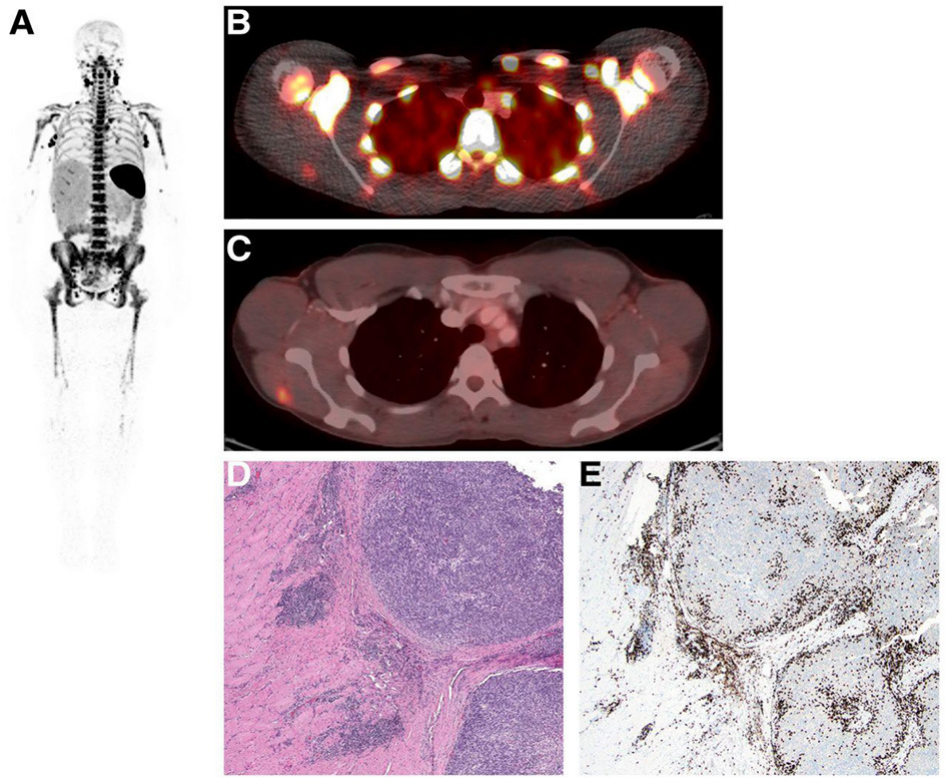
Whole-body immuno-PET imaging with [^89^Zr]Zr-IAB22M2C in a patient at 24 h post-injection. **(A)** Intense uptake is noted in lymph nodes. **(B, C)** Fusion image at 24 h shows [^89^Zr]Zr-IAB22M2C uptake in lesion and deltoid **(B)**, which were also [^18^F]FDG positive **(C)**. **(D)** H&E stained section shows melanoma tumor nodules on the right within skeletal muscle. **(E)** IHC highlights the presence of CD8^+^ T-cells at the periphery and infiltrating tumors [reproduced with permission from original publication Pandit-Taskar et al. (183)].

In the TME and in the systemic circulation, TILs, cytotoxic CD8^+^ and helper CD4^+^ T-cells, play a key role in driving the antitumor immunological responses in immunotherapies. With radiolabeled CD8- and CD4-specific minibodies (72, 180, 181) and cys-diabodies (43, 182), immuno-PET imaging has been proven with the capability to reveal T-cell-enriched tissues, such as the lymph nodes, spleen, and thymus in mouse models. For instance, immuno-PET with ^89^Zr-labeled anti-CD8 cys-diabody has been found to be able to detect the mobilization of CD8-expressing T lymphocytes from the systemic circulation to tumors in syngeneic mouse models when subjected to immunotherapies with an agonistic mAb (anti-CD137/4-1BB), checkpoint blockade mAb (anti–PD-L1), and ACT (182). Recently, a ^89^Zr-labeled anti-CD8 minibody (^89^Zr-Df-IAB22M2C) has advanced to early-phase clinical trials in subjects with primary (183) (Figure 5) and metastatic solid tumors (melanoma, non-small cell lung cancer, and hepatocellular carcinomas) (184). Furthermore, another report using ^64^Cu labeled IAB22M2C has described similar applications in brain tumors (66, 180). Notably, bispecific antibody constructs consisting of two ScFv arms have been seen in this endeavor, one targeting the tumor antigen and the other often targeting CD3 markers on T-cells. However, in such bispecific constructs, the target with the stronger affinity to the radiotracer may likely predominate in the radiotracer's biodistribution. For example, in a first-in-human imaging study with a carcinoembryonic antigen (CEA)/CD3-targeting radiotracer, ^89^Zr-AMG 211, intra- and inter-subject heterogeneous tumor uptake was observed, which was largely dominated by the CD3 arm. As such, the imaging results likely depicted T-cell distribution (166, 185). While the faster clearance and earlier imaging time points seen with mAb fragments are clinically advantageous, their imaging sensitivity and specificity remain to be improved, as their specific binding affinities are inevitably compromised as compared to their mAb counterparts (186).

*In vivo* tracking of CAR T-cells can be realized by direct labeling with ^111^In, ^89^Zr, ^99m^Tc, or ^68^Ga (187–191). However, these techniques face major challenges for longitudinal *in vivo* tracking due to the loss of radiolabels during subsequent passages of CAR T-cells and decay of the radionuclide (192). Moreover, this type of radiolabeling technique cannot distinguish between live and dead CAR T-cells, although the latter are likely to be digested or sequestered in the liver or spleen. Therefore, transduction of CAR T-cells with a protein reporter has been employed for pairing with a well-established PET imaging method. To date, many such reporter/radiotracer pairs have been developed to capture the spatiotemporal expansion of CAR T-cells in preclinical mouse models (51, 178, 193): for instance, PSMA can be paired with [^18^F]F-DCFPyL, a PSMA-specific PET agent (194). PSMA was chosen because of its well-accepted role in theranostic treatment of cancers (195, 196). Another protein of interest is the somatostatin receptor 2 (SSTR2), a G-protein-coupled membrane receptor with basal expression in normal tissues and overexpression in many neuroendocrine tumors (NETs) (197–199). Notably, a recent study has gone one step further to investigate the potential of using the SSTR2 reporter as a suicide switch to destroy the CAR T-cells when they generate toxic AEs. In this approach, a maytansine–octreotate conjugate, PEN-221 (Tarveda), was used for imaging and elimination of CAR T-cells when they became toxic (200). Another interesting study used an engineered antibody against DOTA (1,4,7,10-tetraazacyclododecane-1,4,7,10-tetraacetic acid; DAbR1) for both cell tracking and a potential antibody–drug conjugate (201). DAbR1 contains a single-chain fragment of the anti–lanthanoid-DOTA antibody 2D12.5/G54C fused to the human CD4-transmembrane domain and binds irreversibly to lanthanoid (S)-2-(4-acrylamidobenzyl)-DOTA (AABD) (202) for imaging of DAbR1-positive T-cells when labeled with ^86^Y (201).

While most of these reporter/radiotracer studies are still at the preclinical stages, a successful first-in-human trial tracking CAR T-cells has been reported in the case of a 57-year-old man with grade IV glioblastoma whose autologous CD8^+^ T-cells were genetically engineered to express the herpes simplex virus type 1 thymidine kinase (HSV1-tk) suicide gene for PET imaging with 9-[4-[^18^F]fluoro-3-(hydroxymethyl)butyl]guanine ([^18^F]F-FHBG) (203). This approach was further validated in a subsequent clinical trial with a cohort of six patients with glioblastoma (204). It is noteworthy that, while these studies have set the stage for clinical CAR T-cell imaging, the challenges are also evident in terms of their limited clinical practicality (e.g., extrinsic viral proteins are required and signal-to-noise ratios are suboptimal) (205).

### T-cell activation

**Figure 6:**
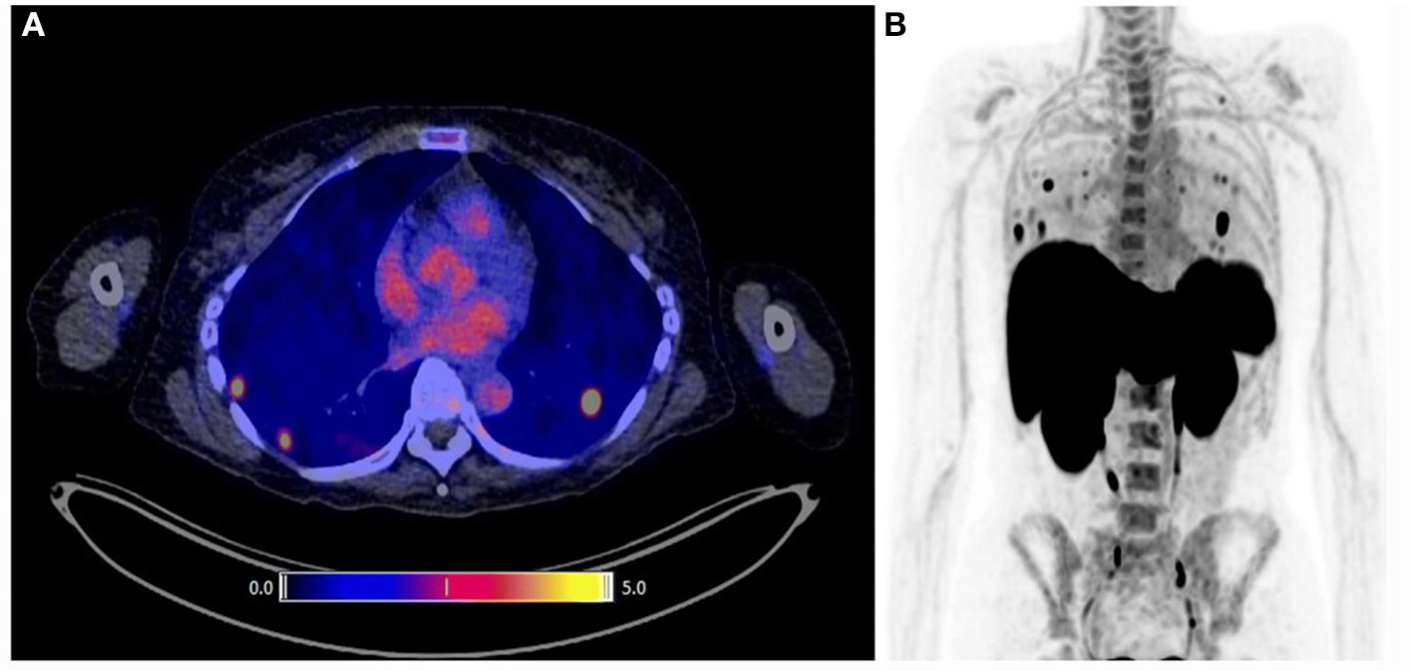
Representative [^18^F]FB-IL2 PET images of human melanoma. **(A)** Transversal PET/CT image of three regions showing high [^18^F]FB-IL2 uptake. **(B)** MIP image of the same patient showing multiple areas of high radiotracer accumulation in the lungs. Reproduced from the open access article in ref. (158) under the Creative Commons license (http://creativecommons.org/licenses/by/4.0/).

While imaging of T-cell lineage markers (e.g., CD3, CD4, and CD8) can provide information regarding mobilization and tumor retention of the T-cells (both CAR T-cell therapies and other T-cells), it is essential to know whether these T-cells are activated. Immuno-PET imaging of proteins specifically upregulated during T-cell activation can function as biomarkers to address this issue (Supplementary Table S4). For instance, inducible T-cell costimulator (ICOS) is a T-cell co-stimulatory molecule upregulated during T-cell activation. Using a ^89^Zr-labeled anti-ICOS mAb, the activation, expansion, and tumor retention of CD19-directed CAR T-cells have been investigated in a mouse model of B-cell lymphoma (44, 206). Absent in resting naïve T-cells, CD134 or OX40 could also be used as T-cell activation markers (207). Furthermore, cytokines generated in response to T-cell activation, such as IFN-γ (160) and IL-2 (Figure 6) (158, 208), have also been reported on for use in immuno-PET imaging of T-cell activation.

Molecular imaging of T-cell distribution and activation is capable of providing an immune signature of ongoing cytotoxic responses or possibly of resistance to immunotherapies (209, 210). Recent reports on a PET agent that targets human granzyme B, ^68^Ga-NOTA-GZP, are noteworthy (209, 211, 212). Granzyme B is a pro-apoptotic serine protease, secreted and activated via granular exocytosis along with perforin by activated cytotoxic T-cells and NK cells. It initiates the target cell death cascade by caspase activation (213, 214). Interestingly, ^68^Ga-NOTA-GZP only targets the active secreted form of the enzyme (biological t_1/2_ = 14 days), making it an ideal candidate for detection of the extent of cytotoxic response, or lack thereof, in the TME (212). Interestingly, PET with ^68^Ga-NOTA-GZP has been found to be able to reveal distinct immune signatures associated with immunoactivation in tumors and tumor-draining lymph nodes.

### Adverse toxic events

CAR T-cell therapies as a class have been found to be associated with certain unique toxicities due to the immunological surge of cytokines that follows the CAR T-cell-based T-cell activation cascade. These AEs are termed CRS and ICANS, which encompass the most notable CAR T-cell toxicities. Additionally, other toxicities such as “on-target, off-tumor” and anaphylactic effects have been reported. Unlike side effects observed with other chemotherapeutics, which are often non-specific, the toxicities observed in CAR T-cell therapy are on-target and reversible in most cases. Minimization of these toxic events is highly desirable in clinical management with CAR T-cell therapies (33).

To date, several pathophysiological mechanisms behind the occurrence of CRS and ICANS have been elucidated in the literature (215–217). Usually, CRS is triggered within days after CAR T-cell activation post-infusion, although delayed AEs may occur up to 3 weeks post-infusion due to prolonged systemic circulation of the CAR T-cells (218). The symptoms of CRS are mainly perpetuated by elevated circulating levels of pro-inflammatory cytokines, including IFN-γ, TNF-α granulocyte-macrophage colony-stimulating factor (GM-CSF), IL-10, IL-1, and IL-6, as well as other inflammatory mediators such as nitric oxide (216, 219). These AEs range from mild (grade 1) flu-like symptoms to more severe (grade 3–4) manifestations, including hypotension, tachycardia, difficulty breathing, hypoxia and capillary leak, hypoalbuminemia, coagulopathy, shock, and, in some rare cases, multiple organ injury/failure, which need immediate medical attention (220–222). CRS-related AEs are in fact quite commonly encountered in patients treated with CAR T-cell therapeutics, with ~53%−93% experiencing different grades of CRS-related AEs, ~13%−14% undergoing severe (≥ grade 3) reactions, and ~20%−50% needing to be transferred to intensive care units (30, 216, 223, 224). These AEs are not limited by disease phenotype (they are seen in lymphomas, leukemias, and even myelomas) or by the type of antigen being targeted, although the CRS AEs and neurotoxicity are more commonly seen with CD19-directed CAR T-cell therapies than others (30, 219, 220, 225). Notably, CRS is not limited to CAR T-cell therapies and may be seen with other agents (215, 226) and immunopathologic conditions that may affect B- and/or T-cell function (216). ICANS is the second major form of toxicity seen with CAR T-cell therapies as well as BiTEs, with symptoms including encephalopathy, aphasia, delirium, tremor, and seizures (225). The pathophysiology of ICANS is still largely unknown as compared to CRS. Under the mechanisms that have been proposed, initial pro-inflammatory cytokine activation mediated by CAR T-cells may result in endothelial activation and increased microvascular permeability, which may lead to disruption of the blood–brain barrier and subsequent passive diffusion of CAR T-cells and cytokines in the central nervous system (CNS). These events may further trigger a positive immunoactivation feedback loop for manifestation of ICANS (216, 217). While they are more commonly seen with CD19-directed CAR T-cells, neurotoxic AEs also occur with other non-CD19-targeting CAR T-cell therapies (225). Although less common than CRS, ICANS is known to occur in ~21%−66% of all patients treated with CAR T-cell therapies, with severe ICANS AEs (≥ grade 3) occurring in ~12%−45% (225, 227). Elevated cytokine levels in CRS may often precede neurotoxic events. However, ICANS may occur concurrently, after CRS has subsided, or even independently of CRS. The symptoms of ICANS may be relatively mild without CRS interplay (216, 217). As CAR T-cell therapies progress toward applications in solid tumors, unique “on-target, off-tumor” forms of toxicity have been encountered. Such toxicities may occur in non-diseased tissues, wherein CAR T-cells primed to attack the tumors overexpressing the target antigen may also affect normal tissues with basal antigen expression (228). In these situations, the expression level of the target antigen on the non-diseased tissues becomes a determinant of the severity of the AE (33, 229). Other rare instances of toxicity include symptoms such as anaphylaxis following an immune response to the CAR (230); in most instances, these occur as a result of the mAb-derived antigen-recognition domains in the CAR structure (231).

Currently, there are no approved preventive measures for these toxicities (232). However, whole-body PET or SPECT imaging has the capability to locate these AEs as they occur anywhere in the body, which can be leveraged to guide therapeutic interventions (233). In clinical practice, [^18^F]FDG-PET plays a role in the management of CAR T-cell therapies, but it cannot differentiate neoplastic disease or other inflammatory events from a hyper-inflammatory episode such as CRS (233). To the best of our knowledge, no immuno-PET approaches have been reported for imaging of these toxicities. As a wide array of cytokines are upregulated at different points during the CRS cascade (216), imaging specificity is difficult to achieve. Another major hurdle is the lack of a suitable mouse model for investigation of CRS events, although two humanized mouse models have been reported for CRS and ICANS that may be useful for proof-of-concept studies (234, 235). To date, sufficient evidence has shown IL-6 serum cytokine levels to be the most significantly elevated during CRS (215, 236, 237). Clinically, tocilizumab, a mAb inhibitor of IL-6 receptor (IL-6R), has therapeutic applications as a first-line agent with corticosteroids to treat grade 2 CRS AEs in patients receiving CAR T-cell therapies (237, 238). Therefore, immuno-SPECT with ^99m^Tc-labeled tocilizumab and optical imaging with Cy7-tagged tocilizumab have been reported for preclinical imaging of myelomas (239–241). Siltuximab is another IL-6 inhibitor mAb that is used as an alternative third-line treatment in patients with CRS and ICANS who are unresponsive to tocilizumab and corticosteroids (220, 242). Both mAbs can be considered for immuno-PET imaging in CRS. IL-1 is another key player generated early in CRS initiation, and Anakinra^®^, a recombinant human IL-1 antagonist, has demonstrated favorable efficacy against CRS and ICANS based on a study in humanized models and early clinical trials (234, 235). While ^18^F and ^99m^Tc radiolabeling methods have been reported for peptides inhibiting IL-1, *in vivo* imaging remains to be evaluated for these probes (243–246). Moreover, immuno-PET imaging with [^89^Zr]Zr-α-IL-1β has been found to be able to detect colonic inflammation in murine dextran sodium sulfate-treated colitic models, which correlates with the severity of the disease (247). Rather than directly targeting individual cytokines mediating CRS or ICANS, targeting of their common upregulated downstream immune checkpoints (indirect targeting) may hold promise for imaging of these toxicities. For instance, PD-L1 is known to be upregulated by multiple cytokines as an inhibitory ligand in the PD-1/PD-L1 checkpoint axis; this synergistic upregulation may provide a strong imaging signal enhancement for sensitive detection (41, 114, 248, 249). As such, the validated methods for immuno-PET imaging of PD-L1 remain to be tested for imaging of these AEs.

Notably, crosstalk between cancer cells and the TME plays an important role in targeted therapies for cancer. Advanced solid malignancies often feature a hypoxic and immunosuppressive TME, which acts as a barrier impairing the effectiveness of therapies. In fact, despite impressive clinical outcomes in patients with advanced R/R B-cell hematological malignancies and multiple myelomas, CAR T-cell therapy faces hurdles in treating solid tumors with an immunosuppressive TME (31, 250). Moreover, the neovasculature encompassing solid tumors often restricts infiltration of the TME by CAR T-cells. TME is a specialized environment consisting of dynamic interplays between varieties of cells in the milieu of aberrant metabolites and cell signals (251, 252). Cancer cells can gain survival advantages by manipulating innate cellular mechanisms, for instance, by hijacking immunomodulation pathways to evade immune surveillance or to escape killing by immunotherapies. Due to the dynamic nature of the TME, it can be morphologically, phenotypically, and functionally heterogeneous across time, subjects, and tumor sites (primary vs. metastases), and even across regions within the same tumor (32, 253). Immuno-PET/-SPECT imaging can be leveraged to non-invasively track dynamic changes in the TME in real-time, thus providing invaluable information for precision treatment strategies.

The “hyper-metabolic” state of aggressive malignancies often produces conditions of nutritional deficit, hypoxia, pH reduction (acidosis due to lactic acid generation post-glycolysis) of the TME, and oxidative stress (37). It is well-known that hypoxic conditions stabilize hypoxia-inducible factors that promote angiogenesis. Recently, it has been found that hypoxia induces immune evasion by upregulating the immune checkpoint proteins [e.g., PD-L1, PD-L2, human leukocyte antigen-G (HLA-G), and soluble CD137] (254, 255), impairs the expansion of CAR T-cells, and reduces immune activation (256). Therefore, recent CAR T-cell therapy strategies have been expanded to include transduction of the CAR design with hypoxia-sensing domains (257) and targeting of antigens upregulated in hypoxia [e.g., carbonic anhydrase IX (CAIX) (258)] in order to improve the therapeutic efficacy (32, 259). Of note, immuno-PET with ^124^I or ^89^Zr-labeled girentuximab, an anti-CAIX mAb, has advanced to clinical trials in patients with renal cell carcinoma (260–262) and urothelial cancers (263), while the ^89^Zr-labeled mAb has demonstrated improved detection sensitivity due to the residualizing properties of the radionuclide (264). Immuno-PET/-SPECT with radiolabeled pH-selective mAbs, which has not yet been reported, might find application in non-invasive assessment of TME acidosis (265).

## Conclusion

Adoptive cell therapy has brought about a paradigm shift in cancer treatment using innovative immunotherapy approaches. Evidently, these novel drugs come with their own unknowns, challenges, and certain unique toxicities. Molecular resistance mechanisms, such as antigen loss and T-cell exhaustion, particularly in the immunosuppressive TME, are still the most significant challenges faced by CAR T-cell therapy. Consequently, there is an urgent unmet clinical need for early identification and tracking of these mechanisms in order to implement timely treatment interventions. In recent years, synergizing of the highly sensitive PET and SPECT functional imaging modalities with anatomical/physiological computed tomography or magnetic resonance imaging has generated a multifaceted, highly sensitive, non-invasive platform for real-time detection of dynamic events in live subjects. To date, this platform has been validated for use in clinical diagnosis and disease management in various diseases and conditions, including cancer. Moreover, recent technological advancements and sophisticated algorithms for SPECT have advanced its capability for simultaneous imaging of two radionuclides with differentiable emission energies, thus enabling non-invasive assessment of the simultaneous occurrences of two biological events (266–268). In addition, recent solid-state detectors and advanced reconstruction algorithms have further improved the sensitivity and spatial resolution of SPECT. As such, we expect to see accelerated progresses in immuno-PET/-SPECT imaging and their applications in immunotherapies. In conjunction with the explosive development of innovative strategies in the realm of spatial-omics (transcriptomics, proteomics, and metabolomics), novel targets will certainly emerge for the future development of more practical immuno-PET/-SPECT imaging methodologies to address the challenges of CAR T-cell therapy (269). To add to this arsenal, deep learning-based radiomic analysis of image features extracted from the vast datasets of available images could further move the field forward.

## Author contributions

OKÖ, PR, and XS: conceptualization. AM, DU, and YY: writing—preparation of original draft. AM, DU, SH, OKÖ, PR, and XS: writing—review and editing. AM and DU: visualization. XS: supervision. All authors have read and agreed to the published version of the manuscript.
