# Supplementary material for: Current and potential roles of immuno-PET/-SPECT in CAR T-cell therapy
Source: Front Med (Lausanne). 2023 Jun 27;10:1199146. doi: 10.3389/fmed.2023.1199146 (PMC10333708; doi:10.3389/fmed.2023.1199146)
Supplement: Supplementary file 1 [file Presentation_1.pdf]

SUPPLEMENTARY INFORMATION

Table S1. Selected tumor antigen targeted immuno-PET/-SPECT methods in use or with potential for noninvasive assessment of antigen loss

Target Antigen	Radiotracer	Imaging Modality	Study species	Disease Model	Reference
**CD20**	[^{64}Cu]Cu-DOTA-rituximab	Immuno-PET	Human	B-cell NHL	Lee, 2023 (82)
	[^{89}Zr]Zr-DFO-rituximab	Immuno-PET	Human	DLBCL	Jauw, 2017 (81)
	[^{64}Cu]Cu-DOTA-rituximab	Immuno-PET	Mouse	human CD20^{+} transgenic mice	Natarajan, 2012 (270)
	[^{89}Zr]Zr-DFO-Ibritumomab (Zevalin) and [^{88}Y]Y-Zevalin	Immuno-PET	Human, Mouse	NHL human subjects; NHL murine tumor graft (Ramos cells)	Perk, 2006 (271)
**CD22**	[^{111}In]In-DOTA-10C6, [^{64}Cu]Cu-DOTA-10C6 (anti-CD22c clone), and [^{125}I]I-anti-CD22c mAbs	Immuno-PET, Immuno-SPECT	Mouse, Dog	Mouse (canine B-cell lymphoma cell line, CLBL-1) xenograft; Canine spontaneous DLBCL	Etienne, 2020 (272)
**CD38**	[^{89}Zr]Zr-DFO-Daratumumab	Immuno-PET	Human, Mouse	Multiple myeloma; Mouse models (OPM2 cells)	Ulaner 2020 (273)
		Immuno-PET	Mouse	Multiple myeloma models (CD38^{+} MM1.S-luciferase cells)	Ghai, 2018 (274)
		Immuno-PET	Mouse	NSCLC (A549, H460, H358 cells) models	Ehlerding, 2017 (275)
**VLA4**	[^{64}Cu]Cu-CB-TE1A1P-LLP2A	Immuno-PET/CT, Immuno-PET/MRI	Human	Multiple myeloma	NCT03804424, Laforest, 2023 (276)

Target Antigen	Radiotracer	Imaging Modality	Study species	Disease Model	Reference
**CD19**	[^{64}Cu]Cu-DOTA-CD19-mAb (murine)	Immuno-PET	Mouse	Multiple sclerosis model (EAE model)	Stevens, 2020 (83)
**PSMA**	[^{89}Zr]Zr-DFO-J591	Immuno-PET	Human	mCRPC	Pandit-Taskar, 2015 (91)
	[^{89}Zr]Zr-huJ591 scFv and [^{89}Zr]Zr-cysteine diabodies	Immuno-PET	Mouse	Prostate cancer [LNCaP (PSMA^{+}) and PC3 (PSMA^{-}) cell lines] xenograft models	Viola-Villegas, 2014 (277)
**EGFR**	[^{89}Zr]Zr-DFO-Cetuximab	Immuno-PET	Human	Colorectal cancer	der Houven, 2015 (84)
	[^{89}Zr]Zr-DFO-Panitumumab	Immuno-PET	Mouse	PDX models of ameloblastoma	Stone, 2022 (86)
**HER2**	[^{89}Zr]Zr-DFO-Trastuzumab	Immuno-PET	Human	mEGC	Lumish 2022 (89)
		Immuno-PET	Human	HER-2^{+} breast cancer	Gebhart, 2016 (87), NCT01565200; Ulaner, 2017 (88), NCT02286843; Dijkers, 2010 (278)
	[^{67}Cu]Cu-NOTA-Pertuzumab	Immuno-SPECT (Theranostic)	Mouse	Tumor (HER2^{+} HCC1954 cells) xenografts	Hao, 2021 (42)
	[^{89}Zr]Zr-DFO-Pertuzumab	Immuno-PET	Human	Breast cancer	Ulaner, 2017 (279), NCT03109977
		Immuno-PET	Mouse	Breast cancer (HER2^{+} BT-474 and HER2^{-} MDA-MB-231 cells) xenografts	Marquez, 2014 (280)
	[^{111}In]In-DTPA-Pertuzumab	Immuno-SPECT	Human	Breast cancer	Lam, 2015 (90), NCT01805908
		Immuno-SPECT	Mouse	Breast cancer (MDA-MB-361 cells) xenografts	McLarty, 2009 (281)

Target Antigen	Radiotracer	Imaging Modality	Study species	Disease Model	Reference
**VEGF**	[^{89}Zr]Zr-DFO-Bevacizumab	Immuno-PET	Human	DIPG	Jansen, 2016 (282)
		Immuno-PET	Human	Metastatic RCC	Oosting, 2015 (92), NCT00831857; van der Stegen, 2014 (283)
		Immuno-PET	Human	Breast cancer	Gaykema 2013 (284)

Abbreviations: CAR, Chimeric antigen receptor; CD, Cluster of differentiation; Cu, Copper; DLBCL, Diffuse large B cell lymphoma; DFO, Deferoxamine; DIPG, Diffuse intrinsic pontine glioma; DOTA, 2,2′,2”,2”'-(1,4,7,10-tetraazacyclododecane-1,4,7,10-tetrayl)tetraacetic acid; DTPA, Diethylenetriaminepentaacetic acid; EAE, Experimental autoimmune encephalomyelitis; EGFR, Epidermal growth factor receptor; HER-2, Human epidermal growth factor receptor 2; In, Indium; mAbs, Monoclonal antibodies; mCRPC, Metastatic castration-resistant prostate cancers; mEGC, Metastatic esophagogastric cancer; NHL, Non-Hodgkin’s lymphoma; NOTA, 1,4,7-Triazacyclononane-1,4,7-triacetic acid; NSCLC, Non-small cell lung cancer; PDX, Patient derived xenograft; Immuno-PET, Immunological positron emission tomography; PSMA, Prostate-specific membrane antigen; RCC, Renal cell carcinoma; Immuno-SPECT, Immunological single-photon emission computed tomography; VEGF, Vascular endothelial growth factor; VLA-4, Very late antigen-4; Y, Yttrium; Zr, Zirconium

Table S2. Selected immuno-PET/-SPECT methods in use or with potential for noninvasive assessment of T-cell exhaustion

Target Antigen*	Imaging Radiotracer	Imaging Modality	Species	Disease Model	Reference
**PD-1**	[^{89}Zr]Zr-DFO-Nivolumab	Immuno-PET	Human	NSCLC	Niemeijer, 2017 (285),<br>Clinical Trial: 2015-004760-11
		Immuno-PET	Mouse (Humanized)	Lung cancer (A549 cells) xenografts	England, 2018 (286)
	[^{89}Zr]Zr-DFO-Pembrolizumab	Immuno-PET	Human	NSCLC	Niemeijer, 2021 (287)
		Immuno-PET	Mouse	Melanoma (A375 cells) xenografts	Natarajan, 2017 (288)
**PD-L1**	[^{89}Zr]Zr-DFO-Atezolizumab	Immuno-PET	Human	Breast cancer, NSCLC, Bladder	Bensch, 2018 (130)<br>NCT02453984 and NCT02478099
		Immuno-PET	Mouse, Human	RCC PDX mouse models and Locally advanced or metastatic RCC patients	Mulgaonkar, 2022 (41);<br>Vento, 2019 (131)<br>NCT04006522
	[^{89}Zr]Zr-DFO-Avelumab	Immuno-PET	Mouse	Breast cancer xenografts (MDA-MB-231 cells)	Li, 2020 (289);<br>Jagoda, 2019 (290)
	[^{89}Zr]Zr-DFO-Durvalumab	Immuno-PET	Human	R/M SCCHN before durvalumab monotherapy	Verhoeff, 2022 (291),<br>NCT03829007
		Immuno-PET	Human	SCCHN	Verhoeff, 2020 (292)
	[^{89}Zr]Zr-DFO-REGN3504	Immuno-PET	Mouse (Humanized)	Xenografts (NCI-H441, MDA-MB-231 and HCC827) and tumor grafts (MC38 exp.PD-L1 cells)	Kelly, 2021 (293)
	[^{89}Zr]Zr-DFO-KN035	Immuno-PET	Mouse	GBM (LN229 cells) xenografts	Li, 2018 (294)
		Immuno-PET	Human	Solid tumors	NCT04977128

Target Antigen*	Imaging Radiotracer	Imaging Modality	Species	Disease Model	Reference
	[^{18}F]F- BMS-986192	Immuno-PET	Human	Metastatic melanomas (brain metastases)	Nienhuis, 2022 (295)
		Immuno-PET	Human	NSCLC	Niemeijer, 2017 (285), Clinical Trial: 2015-004760-11
		Immuno-PET	Human	Advanced-stage NSCLC	Huisman, 2020 (296)
	[^{68}Ga]Ga-NODAGA-BMS-986192	Immuno-PET	Mouse (IC), Non-human primate	Mouse bilateral melanoma (B16F10, wild-type and exp. hPD-L1 cells) tumor grafts	Zhou, 2022 (297)
**CTLA-4**	[^{89}Zr]Zr-DFO-Ipilimumab	Immuno-PET	Human	Metastatic melanomas	NCT03313323
	[^{64}Cu]Cu-DOTA-ipilimumab	Immuno-PET	Mouse	NSCLC (A549, H460, and H358 cells) xenografts	Ehlerding, 2017 (133)
	[^{64}Cu]Cu-DOTA-anti-CTLA-4 mAb (murine)	Immuno-PET	Mouse (IC)	Colon cancer (CT26 cells) tumor grafts	Higashikawa, 2014 (134)
**TIGIT**	[^{64}Cu]Cu-DOTA-anti-mouse-TIGIT mAb, and [^{89}Zr]Zr-DFO-anti-mouse-TIGIT mAb (clone 1G9)	Immuno-PET	Mouse	Melanoma allografts (B16F10 cells) and tumor xenografts (HeLa TIGIT$^{+/-}$ cells)	Shaffer, 2021 (138)
	[^{68}Ga]Ga-NOTA-GP12 antagonist peptide	Immuno-PET	Mouse, Human	Melanoma SubQ and pulmonary metastases models (B16F10 cells); Human subjects with lung adrenocarcinoma	Wang, 2022 (298)
**LAG-3**	[^{89}Zr]Zr-BI 754111	Immuno-PET	Human	NSCLC, Head and Neck neoplasms	Meidema, 2023 (140) NCT03780725
	[^{89}Zr]Zr-DFO-REGN3767	Immuno-PET	Mouse	Tumor grafts (MC38 exp. hLAG-3 cells) and xenografts (Raji exp. hPBMCs)	Kelly, 2018 (299)

Target Antigen*	Imaging Radiotracer	Imaging Modality	Species	Disease Model	Reference
**TIM-3**	[^{64}Cu]Cu-NOTA-RMT3-23	Immuno-PET	Mouse (IC)	Mouse melanoma (B16F10 cells)	Wei W, 2020 (141)

*These target antigens can also be leveraged to image toxicities seen CAR T-cell therapies.

Abbreviations: CAR, Chimeric antigen receptor; Cu, Copper; CTLA-4, Cytotoxic T-lymphocyte–associated antigen 4; DFO, Deferoxamine; DOTA, 2,2′,2”,2”'-(1,4,7,10-tetraazacyclododecane-1,4,7,10-tetrayl)tetraacetic acid; exp., Expressing; GBM, Glioblastoma; hPBMC, Human peripheral blood mononuclear cell; IC, Immunocompetent; LAG-3, Lymphocyte activating gene 3; mAb, Monoclonal antibody; NODAGA, 2-[1,4,7-Triazacyclononan-1-yl-4,7-bis(tBu-ester)]-1,5-pentanedioic acid; NOTA, 1,4,7-Triazacyclononane-1,4,7-triacetic acid; NSCLC, Non-small cell lung cancer; PD-1, Programmed cell death-1; PD-L1, Programmed cell death ligand-1; PDX, Patient derived xenograft; Immuno-PET, Immunological positron emission tomography; RCC, Renal cell carcinoma; R/M, Recurrent or metastatic; TIGIT, T-cell immunoglobulin and immunoreceptor tyrosine-based inhibitory motif domain; SCCHN, Squamous cell carcinoma of the head and neck; SubQ, Subcutaneous; TIM-3, T-cell immunoglobulin mucin-3; Zr, Zirconium

Table S3. Selected PET or SPECT methods in use or with potential for noninvasive assessment of T-cell distribution

Target	Imaging Radiotracer	Imaging Modality	Species	Disease Model	Reference
**CD8**	[^{64}Cu]Cu-NOTA-IAB22M2C minibody	Immuno-PET	Mouse (Humanized)	PBMC HIS mouse model, GBM PDX bearing HIS model	Nagle, 2021 (180)
	[^{89}Zr]Zr-DFO-IAB22M2C minibody	Immuno-PET	Mouse	Colorectal cancer (LS1034 cells) xenografts;<br>Treatment: activated/expanded human T-cells + PF-07062119 (CD3 and guanylyl cyclase C targeted BsAb)	Maresca, 2021 (300)
		Immuno-PET	Mouse (Humanized)	MKN-45 and HeLa cervical cancer xenografts;<br>Treatments: CEA-TCB/CEA-4-1BBL and single-agent FOLR1-TCB, respectively	Griessinger, 2020 (301)
		Immuno-PET	Human	Solid tumors	NCT03802123
		Immuno-PET	Human	Melanomas, Lung cancer, HCC	NCT03107663, Pandit-Taskar, 2020 (183)
	[^{64}Cu]Cu-NOTA-anti-mouse CD8a antibody	Immuno-PET	Mouse (IC)	Colon cancer (CT26 cells) tumor grafts	Kristensen, 2020 (302)
	[^{89}Zr]Zr-VHH-X118-PEG20 nanobody	Immuno-PET	Mouse	Influenza A virus WSN/33 infection	Rothlauf, 2021 (303)
	[^{89}Zr]Zr-malDFO-169 cDb cys-diabody	Immuno-PET	Mouse	Mouse orthotopic syngeneic (GSC005 cells) glioma tumors; intracranial immunotherapy with oncolytic HSV M002	Kasten, 2021 (304)

Target	Imaging Radiotracer	Imaging Modality	Species	Disease Model	Reference
		Immuno-PET	Mouse	Colon cancer (CT26 cells) tumor grafts;<br>Treatment: anti-CD137 or anti–PD-L1 mAb	Tavaré, 2016 (182)
	$[^{89}Zr]$Zr-malDFO-2.43 cDb cys diabody	Immuno-PET	Mouse (IC)	Hematopoietic stem cell transplantation model	Tavaré, 2015 (305)
**CD4**	$[^{89}Zr]$Zr-malDFO-GK1.5 cDb cys diabody	Immuno-PET	Mouse (IC)	Hematopoietic stem cell transplantation model	Tavaré, 2015 (305)
**CD3**	$[^{89}Zr]$Zr-anti-CD3,<br>$[^{89}Zr]$Zr-anti-IFN-gamma	Immuno-PET	Mouse	Mammary (neu+TUBO cells) tumors + HER2/neu DNA vaccination	Gibson, 2018 (160)
**Direct T-cell labeling**	$[^{89}Zr]$Zr-oxine	Immuno-PET	Mouse	Xenograft models: GBM orthotopic (PBT030-2 ffLuc-positive glioma cells) + intra-cranially delivered IL13Rα2-CAR T-cells; SubQ prostate (PC3-PSCA) tumors + IV delivered PSCA-CAR T-cells	Weist, 2018 (188)
**CAR T-cells co-transduced with CD19 + PSMA-targeted variants (CD19-tPSMA$^{(N9del)}$)**	$[^{18}F]$DCFPyL	Immuno-PET	Mouse (IC)	Xenograft models (Nalm6-eGFP-fLuc leukemia cells)	Minn, 2019 (194)
**SSTR2 targeted CAR T-cells**	$[^{68}Ga]$Ga-DOTATOC	Immuno-PET	Mouse (IC)	Xenograft models (SSTR2-exp. Jurkat cells)	Vedvyas, 2016 (178)
**CD19-CAR T-cells co-transduced with DAbR + GFP**	$[^{86}Y]$Y-AABD (immuno-PET imaging), and $[^{177}Lu]$Lu-AABD (immuno-SPECT imaging and CAR ablation)	Immuno-PET, Immuno-SPECT	Mouse (IC)	U373.eGFP.ffLuc cells + injection of CD19 directed CAR-DAbR1	Krebs, 2018 (201)

Target	Imaging Radiotracer	Imaging Modality	Species	Disease Model	Reference
**CD34-TK transduced allogeneic donor T-cells (HSV1-tk/CD34 T-cells)**	[^{18}F]FHBG	Immuno-PET	Human	Leukemia, NHL, Hodgkins Disease, myelodysplastic syndrome, multiple myeloma	NCT00871702
**CD8^{+} T-cells co-expressing HSV1-TK + IL-13 zetakine CAR (HSV1-tk/GRm13Z40 CAR-T)**	[^{18}F]FHBG	Immuno-PET	Human	High grade malignant glioma	Keu, 2017 (204), NCT00730613, NCT01082926
**PSMA-targeted T-cells co-expressing HSV-tk + P28z receptor (HSV1-tk/P28z CAR-T)**	[^{18}F]-FIAU	Immuno-PET	Human	CMPC	Slovin, 2013 (306), NCT01140373

Abbreviations: AABD, (S)-2-(4-acrylamidobenzyl)-DOTA; BsAb, Bi-specific antibody; CAR, Chimeric antigen receptor; cDb, cys diabody; CD, cluster of differentiation; CMPC, Castrate metastatic prostate cancer; CEA-TCB/CEA-4-1BBL, Carcinoembryonic antigen T-cell bispecific antibody (RG7802) and CEA-targeted 4-1BB agonist; Cu, Copper; DAbR1, DOTA antibody reporter 1; DOTATOC, 1,4,7,10-tetraazacyclododecane-N^{I},N^{II},N^{III},N^{IIII}-tetraacetic acid (D)-Phe^{1}-thy^{3}-octreotide; DFO, Deferoxamine; DOTA, 2,2′,2”,2”'-(1,4,7,10-tetraazacyclododecane-1,4,7,10-tetrayl)tetraacetic acid; exp., Expressing; [^{18}F]DCFPyL, 2-(3-{1-carboxy-5-[(6-[^{18}F] fluoro-pyridine-3-carbonyl)-amino]-pentyl}-ureido)-pentanedioic; [^{18}F]-FHBG, 9-(4-[^{18}F]-Fluoro-3-[hydroxymethyl]butyl)guanine; ffLuc, Firefly luciferase; [^{18}F]-FIAU, 2'-deoxy-2'-[^{18}F]-fluoro-1-beta-D-arabinofuranosyl-5-iodouracil; FOLR1-TCB, Folate receptor 1-T-cell bispecific antibody; GBM, Glioblastoma; HCC, Hepatocellular carcinoma; HER-2, Human epidermal growth factor receptor 2; HIS, Human immune system; hPBMC, Human peripheral blood mononuclear cell; HSV, Herpes simplex virus; HSV1-tk, Herpes simplex virus-1 thymidine kinase; IC, Immunocompetent; IFN, Interferon; IL-13, Interleukin-13; IL13Rα2, Interleukin 13 receptor alpha 2; Lu, Lutetium; malDFO, Maleimide-deferoxamine; mAb, Monoclonal antibody; NOTA, 1,4,7-Triazacyclononane-1,4,7-triacetic acid; SSTR2, Somatostatin receptor 2; PD-L1, Programmed cell death ligand-1; PDX, Patient derived xenograft; PSCA, Prostate stem cell antigen; Immuno-PET, Immunological positron emission tomography; Immuno-SPECT, Immunological single-photon emission computed tomography; VHH, Variable Heavy domain of Heavy chain; Y, Yttrium; Zr, Zirconium

Table S4. Selected PET or SPECT methods in use or with potential for noninvasive assessment of T-cell activation

Target Antigen	Imaging Radiotracer	Imaging Modality	Species	Disease Model	Reference
**ICOS**	[^{89}Zr]Zr-DFO-anti-mouse-ICOS mAb (clone:7E.17G9)	Immuno-PET	Mouse	Lung cancer tumor	Xiao, 2020 (206)
		Immuno-PET	Mouse	B-cell lymphoma model (A20 cells)	Simonetta, 2021 (44)
**Interferon gamma***	[^{89}Zr]Zr-anti-CD3, and [^{89}Zr]Zr-anti-IFN-gamma	Immuno-PET	Mouse	Mammary (neu^{+}TUBO cells) tumors + HER2/neu DNA vaccination	Gibson, 2018 (160)
**OX40**	[^{64}Cu]Cu-DOTA-mAbOX40 (Murine)	Immuno-PET	Mouse (IC)	Major MHC-mismatch model of acute GvHD	Alam, 2020 (307)
		Immuno-PET	Mouse (IC)	in situ CpG oligodeoxynucleotide vaccination model with B-cell lymphoma dual tumor grafts (A20 cells)	Alam, 2018 (207)
		Immuno-PET	Mouse (IC)	Major MHC-mismatch model of acute GvHD	Simonetta, 2018 (308)
**Granzyme B**	[^{68}Ga]Ga-NOTA-GZP peptide (Murine)	Immuno-PET	Mouse (IC)	Colon cancer (CT26 cells) tumor grafts; Immunotherapy: Anti-mouse PD-1, anti-mouse CTLA-4	Larimer 2017 (211)
		Immuno-PET	Mouse (IC)	Colon cancer (CT26 and MC38 cells) tumor grafts; Immunotherapy: Anti-mouse PD-1, anti-mouse CTLA-4, anti-mouse TIM-3	Larimer 2019 (212)
		Immuno-PET	Mouse (IC)	Colon cancer (CT26 and MC38 cells) tumor grafts; Immunotherapy: Anti-mouse PD-1, anti-mouse CTLA-4	LaSalle, 2020 (209)

Target Antigen	Imaging Radiotracer	Imaging Modality	Species	Disease Model	Reference
**IL-2***	$[^{18}F]$FB-IL2	Immuno-PET	Human	ICI treatment-naïve Metastatic melanoma (Stage IV)	van de Donk, 2021 (158), NCT02922283
	$[^{18}F]$AlF-RESCA-IL2	Immuno-PET	Human	Metastatic solid tumors	NCT05471271
	$[^{68}Ga]$Ga-NODAGA-IL2, $[^{18}F]$AlF-RESCA-IL2, $[^{18}F]$FB-IL2	Immuno-PET	Mouse (ID)	Mice inoculated with activated hPBMCs xenografts	van der Veen, 2020 (208)
	$[^{18}F]$FB-IL2	Immuno-PET	Mouse (ID), Rats (IC)	Animals inoculated with PHA activated hPBMCs	Hartimath, 2018 (309)
**Deoxyguanosine kinase**	$[^{18}F]$F-AraG	Immuno-PET	Mouse (IC)	Syngeneic tumor models (MC38, CT26, LLC, A9F1, 4T1, and B16F10 cells)	Levi, 2021 (310)
		Immuno-PET	Mouse (IC)	Immunogenic KPC ($Kras^{Lox\text{-}STOP\text{-}Lox\text{-}G12D}$, $p53^{fl/fl}$, PDX1-Cre cells) pancreatic tumors exp. inversion-induced joined neoantigen (NINJA)	Cavaliere, 2021 (311)
**Deoxycytidine kinase**	$[^{18}F]$-FAC	Immuno-PET	Human	PDAC	NCT05141643
	$[^{18}F]$-FAC, $[^{18}F]$-CFA	Immuno-PET	Mouse (IC and ID)	EAE model	Chen, 2020 (312)
	$[^{18}F]$-FAC	Immuno-PET	Mouse (IC and ID)	ConA treatment to induce Autoimmune hepatitis	Salas, 2018 (313)

Target Antigen	Imaging Radiotracer	Imaging Modality	Species	Disease Model	Reference
	[^{18}F]CFA and [^{18}F]-AraG	Immuno-PET	Mouse (IC), Human	Mouse xenograft model (CEM-EYFP and CEM-CDA lymphoblastic cells); Paraganglioma patient	Kim, 2016 (314)

*These target antigens can also be investigated to image toxicities seen CAR T-cell therapies. CAR, Chimeric antigen receptor; CD, cluster of differentiation; ConA, Concanavlin A; Cu, Copper; CTLA-4, Cytotoxic T-lymphocyte–associated antigen 4; DFO, Deferoxamine; DNA, Deoxyribonucleic acid; DOTA, 2,2′,2”,2‴-(1,4,7,10-tetraazacyclododecane-1,4,7,10-tetrayl)tetraacetic acid; EAE, Experimental autoimmune encephalomyelitis; exp., Expressing; [^{18}F]-AlF-RESCA-IL2, [^{18}F]-fluoride-(restrained complexing agent)-IL2; [^{18}F]F-AraG, 2′-deoxy-2′-[^{18}F]fluoro-9-β-D-arabinofuranosylguanine; [^{18}F]-CFA, 2-chloro-2'-deoxy-2'-[^{18}F]fluoro-9-β-d-arabinofuranosyl-adenine; [^{18}F]-FAC, [^{18}F]-1-(2′-deoxy-2′-fluoro-arabinofuranosyl)cytosine; [^{18}F]FB-IL2, N-(4-[^{18}F]-fluorobenzoyl)-interleukin-2; GBM, Glioblastoma; GvHD, Graft versus host disease; hPBMC, Human peripheral blood mononuclear cell; IC, Immunocompetent; ICI, immune checkpoint inhibitor; ICOS, Inducible T-cell co-stimulator; ID, immunodeficient; IFN, interferon; IL-2, interleukin-2; mAb, Monoclonal antibody; MHC, Major histocompatibility complex; NODAGA, 2-[1,4,7-Triazacyclononan-1-yl-4,7-bis(tBu-ester)]-1,5-pentanedioic acid; NOTA, 1,4,7-Triazacyclononane-1,4,7-triacetic acid; PDAC, pancreatic ductal adenocarcinoma; PD-1, Programmed cell death-1; PD-L1, Programmed cell death ligand-1; PDX, Patient derived xenograft; Immuno-PET, Immunological positron emission tomography; PHA, Phytohaemagglutinin; TIM-3, T-cell immunoglobulin mucin-3; Zr, Zirconium

Supplemental References:

270. Natarajan A, Gowrishankar G, Nielsen CH, Wang S, Iagaru A, Goris ML, et al. Positron emission tomography of ^{64}Cu-DOTA-rituximab in a transgenic mouse model expressing human CD20 for clinical translation to image NHL. *Mol Imaging Biol* (2012) 14(5):608-16. doi: 10.1007/s11307-011-0537-8.

271. Perk LR, Visser OJ, Stigter-van Walsum M, Vosjan MJWD, Visser GWM, Zijlstra JM, et al. Preparation and evaluation of ^{89}Zr-Zevalin for monitoring of ^{90}Y-Zevalin biodistribution with positron emission tomography. *Eur J Nuc Med Mol Imag* (2006) 33(11):1337-45. doi: 10.1007/s00259-006-0160-0.

272. Etienne F, Berthaud M, Nguyen F, Bernardeau K, Maurel C, Bodet-Milin C, et al. SPECT-CT imaging of dog spontaneous diffuse large B-cell lymphoma targeting CD22 for the implementation of a relevant preclinical model for human. *Front Oncol* (2020) 10. doi: 10.3389/fonc.2020.00020.

273. Ulaner GA, Sobol NB, O'Donoghue JA, Kirov AS, Riedl CC, Min R, et al. CD38-targeted immuno-PET of multiple myeloma: From xenograft models to first-in-human imaging. *Radiology* (2020) 295(3):606-15. doi: 10.1148/radiol.2020192621.

274. Ghai A, Maji D, Cho N, Chanswangphuwana C, Rettig M, Shen D, et al. Preclinical development of CD38-targeted [^{89}Zr]Zr-DFO-daratumumab for imaging multiple myeloma. *J Nuc Med* (2018) 59(2):216-22. Epub 2017/10/14. doi: 10.2967/jnumed.117.196063.

275. Ehlerding EB, England CG, Jiang D, Graves SA, Kang L, Lacognata S, et al. CD38 as a PET imaging target in lung cancer. *Mol Pharmaceut* (2017) 14(7):2400-6. Epub 2017/06/08. doi: 10.1021/acs.molpharmaceut.7b00298.

276. Laforest R, Ghai A, Fraum TJ, Oyama R, Frye J, Kaemmerer H, et al. First-in-Humans evaluation of safety and dosimetry of ^{64}Cu-LLP2A for PET imaging. *J Nuc Med* (2023) 64(2):320-8. doi: 10.2967/jnumed.122.264349.

277. Viola-Villegas NT, Sevak KK, Carlin SD, Doran MG, Evans HW, Bartlett DW, et al. Noninvasive imaging of PSMA in prostate tumors with ^{89}Zr-labeled huJ591 engineered antibody fragments: The faster alternatives. *Mol Pharmaceut* (2014) 11(11):3965-73. doi: 10.1021/mp500164r.

278. Dijkers EC, Oude Munnink TH, Kosterink JG, Brouwers AH, Jager PL, de Jong JR, et al. Biodistribution of ^{89}Zr-trastuzumab and PET imaging of HER2-positive lesions in patients with metastatic breast cancer. *Clin Pharmacol Ther* (2010) 87(5):586-92. Epub 2010/04/02. doi: 10.1038/clpt.2010.12.

279. Ulaner GA, Lyashchenko SK, Riedl C, Ruan S, Zanzonico PB, Lake D, et al. First-in-human HER2-targeted imaging using ^{89}Zr-pertuzumab PET/CT: Dosimetry and clinical application in patients with breast cancer. *J Nuc Med* (2017):jnumed.117.202010. doi: 10.2967/jnumed.117.202010.

280. Marquez BV, Ikotun OF, Zheleznyak A, Wright B, Hari-Raj A, Pierce RA, et al. Evaluation of ^{89}Zr-pertuzumab in breast cancer xenografts. *Mol Pharmaceut* (2014) 11(11):3988-95. doi: 10.1021/mp500323d.

281. McLarty K, Cornelissen B, Cai Z, Scollard DA, Costantini DL, Done SJ, et al. Micro-SPECT/CT with ^{111}In-DTPA-pertuzumab sensitively detects trastuzumab-mediated HER2 downregulation and tumor response in athymic mice bearing MDA-MB-361 human breast cancer xenografts. *J Nuc Med* (2009) 50(8):1340-8. doi: 10.2967/jnumed.109.062224.

282. Jansen MH, Veldhuijzen van Zanten SEM, van Vuurden DG, Huisman MC, Vugts DJ, Hoekstra OS, et al. Molecular drug imaging: ^{89}Zr-bevacizumab PET in children with diffuse intrinsic pontine glioma. *J Nuc Med* (2017) 58(5):711-6. Epub 2016/10/22. doi: 10.2967/jnumed.116.180216.

283. van der Stegen SJ, Hamieh M, Sadelain M. The pharmacology of second-generation chimeric antigen receptors. *Nat Rev Drug Discov* (2015) 14(7):499-509.

284. Gaykema SB, Brouwers AH, Lub-de Hooge MN, Pleijhuis RG, Timmer-Bosscha H, Pot L, et al. ^{89}Zr-bevacizumab PET imaging in primary breast cancer. *J Nuc Med* (2013) 54(7):1014-8. Epub 2013/05/09. doi: 10.2967/jnumed.112.117218.

285. Niemeijer A-LN, Smit EF, Dongen GaMSv, Windhorst AD, Huisman MC, Hendrikse NH, et al. Whole body PD-1 and PD-L1 PET with ^{89}Zr-nivolumab and ^{18}F- BMS-986192 in patients with NSCLC. *J Clin Oncol* (2017) 35(15_suppl):e20047-e. doi: 10.1200/JCO.2017.35.15_suppl.e20047.

286. England CG, Jiang D, Ehlerding EB, Rekoske BT, Ellison PA, Hernandez R, et al. ^{89}Zr-labeled nivolumab for imaging of T-cell infiltration in a humanized murine model of lung cancer. *Eur J Nuc Med Mol Imag* (2018) 45(1):110-20. Epub 2017/08/20. doi: 10.1007/s00259-017-3803-4.

287. Niemeijer A-LN, Oprea Lager DE, Huisman MC, Hoekstra OS, Boellaard R, van de Veen B, et al. First-in-human study of ^{89}Zr-pembrolizumab PET/CT in patients with advanced stage non-small-cell lung cancer. *J Nuc Med* (2021):jnumed.121.261926. doi: 10.2967/jnumed.121.261926.

288. Natarajan A, Mayer AT, Reeves RE, Nagamine CM, Gambhir SS. Development of novel immunoPET tracers to image human PD-1 checkpoint expression on tumor-infiltrating lymphocytes in a humanized mouse model. *Mol Imaging Biol* (2017) 19(6):903-14. Epub 2017/03/02. doi: 10.1007/s11307-017-1060-3.

289. Li M, Ehlerding EB, Jiang D, Barnhart TE, Chen W, Cao T, et al. In vivo characterization of PD-L1 expression in breast cancer by immuno-PET with ^{89}Zr-labeled avelumab. *Am J Transl Res* (2020) 12(5):1862-72. Epub 2020/06/09.

290. Jagoda EM, Vasalatiy O, Basuli F, Opina ACL, Williams MR, Wong K, et al. Immuno-PET imaging of the programmed cell death-1 ligand (PD-L1) using a zirconium-89 labeled therapeutic antibody, avelumab. *Mol Imaging* (2019) 18:1536012119829986. Epub 2019/05/03. doi: 10.1177/1536012119829986.

291. Verhoeff SR, van de Donk PP, Aarntzen E, Oosting SF, Brouwers AH, Miedema IHC, et al. ^{89}Zr-DFO-durvalumab PET/CT before durvalumab treatment in patients with recurrent or metastatic head and neck cancer. *J Nuc Med* (2022) 63(10):1523-30. Epub 2022/05/06. doi: 10.2967/jnumed.121.263470.

292. Verhoeff S, Donk PPvd, Aarntzen EHJG, Miedema IHC, Oosting S, Voortman J, et al. ^{89}Zr-durvalumab PD-L1 PET in recurrent or metastatic (R/M) squamous cell carcinoma of the head and neck. *J Clin Oncol* (2020) 38(15_suppl):3573-. doi: 10.1200/JCO.2020.38.15_suppl.3573.

293. Kelly MP, Makonnen S, Hickey C, Arnold TC, Giurleo JT, Tavaré R, et al. Preclinical PET imaging with the novel human antibody ^{89}Zr-DFO-REGN3504 sensitively detects PD-L1 expression in tumors and normal tissues. *J ImmunoTher Cancer* (2021) 9(1):e002025. doi: 10.1136/jitc-2020-002025.

294. Li D, Cheng S, Zou S, Zhu D, Zhu T, Wang P, et al. Immuno-PET imaging of ^{89}Zr labeled anti-PD-L1 domain antibody. *Mol Pharmaceut* (2018) 15(4):1674-81. Epub 2018/03/06. doi: 10.1021/acs.molpharmaceut.8b00062.

295. Nienhuis PH, Antunes IF, Glaudemans A, Jalving M, Leung D, Noordzij W, et al. ^{18}F-BMS986192 PET imaging of PD-L1 in metastatic melanoma patients with brain metastases treated with immune checkpoint inhibitors: A pilot study. *J Nuc Med* (2022) 63(6):899-905. Epub 2021/09/11. doi: 10.2967/jnumed.121.262368.

296. Huisman MC, Niemeijer AN, Windhorst AD, Schuit RC, Leung D, Hayes W, et al. Quantification of PD-L1 expression with ^{18}F-BMS-986192 PET/CT in patients with advanced-stage non-small cell lung cancer. *J Nuc Med* (2020) 61(10):1455-60. Epub 2020/02/16. doi: 10.2967/jnumed.119.240895.

297. Zhou H, Bao G, Wang Z, Zhang B, Li D, Chen L, et al. PET imaging of an optimized anti-PD-L1 probe ^{68}Ga-NODAGA-BMS986192 in immunocompetent mice and non-human primates. *EJNMMI Res* (2022) 12(1):35. doi: 10.1186/s13550-022-00906-x.

298. Wang X, Zhou M, Chen B, Liu H, Fang J, Xiang S, et al. Preclinical and exploratory human studies of novel ^{68}Ga-labeled D-peptide antagonist for PET imaging of TIGIT expression in cancers. *Eur J Nuc Med Mol Imag* (2022) 49(8):2584-94. doi: 10.1007/s00259-021-05672-x.

299. Kelly MP, Tavare R, Giurleo JT, Makonnen S, Hickey C, Danton MA, et al. Abstract 3033: Immuno-PET detection of LAG-3 expressing intratumoral lymphocytes using the zirconium-89 radiolabeled fully human anti-LAG-3 antibody REGN3767. *Cancer Res* (2018) 78(13_Supplement):3033-. doi: 10.1158/1538-7445.Am2018-3033.

300. Maresca KP, Chen J, Mathur D, Giddabasappa A, Root A, Narula J, et al. Preclinical evaluation of ^{89}Zr-Df-IAB22M2C PET as an imaging biomarker for the development of the GUCY2C-CD3 bispecific PF-07062119 as a T cell engaging therapy. *Mol Imaging Biol* (2021) 23(6):941-51. doi: 10.1007/s11307-021-01621-0.

301. Griessinger CM, Olafsen T, Mascioni A, Jiang ZK, Zamilpa C, Jia F, et al. The PET-Tracer ^{89}Zr-Df-IAB22M2C enables monitoring of intratumoral CD8 T-cell infiltrates in

tumor-bearing humanized mice after T-cell bispecific antibody treatment. *Cancer Res* (2020) 80(13):2903-13. doi: 10.1158/0008-5472.Can-19-3269.

302. Kristensen LK, Christensen C, Alfsen MZ, Cold S, Nielsen CH, Kjaer A. Monitoring CD8a+ T cell responses to radiotherapy and CTLA-4 blockade using [^{64}Cu]NOTA-CD8a PET imaging. *Mol Imaging Biol* (2020) 22(4):1021-30. doi: 10.1007/s11307-020-01481-0.

303. Rothlauf PW, Li Z, Pishesha N, Xie YJ, Woodham AW, Bousbaine D, et al. Noninvasive immuno-PET imaging of CD8+ T cell behavior in influenza A virus-infected mice. *Front Immunol* (2021) 12. doi: 10.3389/fimmu.2021.777739.

304. Kasten BB, Houson HA, Coleman JM, Leavenworth JW, Markert JM, Wu AM, et al. Positron emission tomography imaging with ^{89}Zr-labeled anti-CD8 cys-diabody reveals CD8+ cell infiltration during oncolytic virus therapy in a glioma murine model. *Sci Rep* (2021) 11(1):15384. doi: 10.1038/s41598-021-94887-x.

305. Tavaré R, McCracken MN, Zettlitz KA, Salazar FB, Olafsen T, Witte ON, et al. Immuno-PET of murine T cell reconstitution postadoptive stem cell transplantation using anti-CD4 and anti-CD8 cys-diabodies. *J Nuc Med* (2015) 56(8):1258-64. doi: 10.2967/jnumed.114.153338.

306. Slovin SF, Wang X, Hullings M, Arauz G, Bartido S, Lewis JS, et al. Chimeric antigen receptor (CAR+) modified T cells targeting prostate-specific membrane antigen (PSMA) in patients (pts) with castrate metastatic prostate cancer (CMPC). *J Clin Oncol* (2013) 31(6_suppl):72-. doi: 10.1200/jco.2013.31.6_suppl.72.

307. Alam IS, Simonetta F, Scheller L, Mayer AT, Murty S, Vermesh O, et al. Visualization of activated T cells by OX40-immunoPET as a strategy for diagnosis of acute graft-versus-host disease. *Cancer Res* (2020) 80(21):4780-90. Epub 2020/09/08. doi: 10.1158/0008-5472.CAN-20-1149.

308. Simonetta F, Alam IS, Mayer AT, Murty S, Vermesh O, Hirai T, et al. Tracking T cell activation by OX40 immuno-PET: A novel strategy for imaging of graft versus host disease. *Blood* (2018) 132:4527. doi: https://doi.org/10.1182/blood-2018-99-116483.

309. Hartimath SV, Manuelli V, Zijlma R, Signore A, Nayak TK, Freimoser-Grundschober A, et al. Pharmacokinetic properties of radiolabeled mutant Interleukin-2v: A PET imaging study. *Oncotarg* (2018) 9(6).

310. Levi J, Goth S, Huynh L, Lam T, Huynh TL, Schulte B, et al. ^{18}F-AraG PET for CD8 profiling of tumors and assessment of immunomodulation by chemotherapy. *J Nuc Med* (2021) 62(6):802-7. doi: 10.2967/jnumed.120.249078.

311. Cavaliere A, Foster G, Zheng MQ, Li Z, Huang H, Levi J, et al. PET imaging of activated T cells in pancreatic tumors with [^{18}F]F-AraG. *J Nuc Med* (2021) 62(supplement 1):1281-.

312. Chen BY, Ghezzi C, Villegas B, Quon A, Radu CG, Witte ON, et al. ^{18}F-FAC PET visualizes brain-infiltrating leukocytes in a mouse model of multiple sclerosis. *J Nuc Med* (2020) 61(5):757-63. doi: 10.2967/jnumed.119.229351.

313. Salas JR, Chen BY, Wong A, Cheng D, Van Arnam JS, Witte ON, et al. ^{18}F-FAC PET selectively images liver-infiltrating CD4 and CD8 T cells in a mouse model of autoimmune hepatitis. *J Nuc Med* (2018) 59(10):1616-23. Epub 2018/04/26. doi: 10.2967/jnumed.118.210328.

314. Kim W, Le TM, Wei L, Poddar S, Bazzy J, Wang X, et al. [^{18}F]CFA as a clinically translatable probe for PET imaging of deoxycytidine kinase activity. *Proc Natl Acad Sci* (2016) 113(15):4027-32. doi: doi:10.1073/pnas.1524212113.
